# Comparative Characterization of Odor-Active Compounds and Fatty Acids in Five Pork Cuts

**DOI:** 10.3390/molecules31203642

**Published:** 2026-10-12

**Authors:** Mingguang Yu, Ting Li, Liran Wang, Liang Zhuang, Yunxia Luan

**Affiliations:** 1 Institute of Quality Standard and Testing Technology, Beijing Academy of Agriculture and Forestry Sciences, Beijing 100097, China; a1402010411@163.com; 2 Key Laboratory of Geriatric Nutrition and Health, Ministry of Education, Beijing Technology and Business University, Beijing 100048, China; liting120902@163.com; 3 Zhongke Yuansheng Biotechnology (Tianjin) Co., Ltd., Tianjin 300450, China; wangliran1990@outlook.com; 4 Institute of Agri-food Processing and Nutrition, Beijing Academy of Agriculture and Forestry Sciences, Beijing 100097, China

**Keywords:** pork cuts, odor-active compounds, SAFE-GC-O-QTOF, fatty acid, sensory evaluation

## Abstract

Characterizing flavor differences among pork cuts is important for understanding their quality characteristics and supporting cut-specific processing and product development. Five pork cuts were therefore systematically characterized using sensory evaluation, SAFE-GC-O-QTOF, aroma extract dilution analysis, quantitative analysis, odor activity values (OAVs), fatty acid profiling, and multivariate statistical analysis. Sensory results showed that different cuts exhibited distinct odor profiles. Specifically, pork belly had more pronounced meaty and fatty aromas, while pork ham exhibited stronger hard-boiled egg and metallic notes. A total of 62 odor-active compounds (OACs) were identified, of which 43 were quantified, with total OAC concentrations ranging from 2471 to 10,954 μg/kg. Combining OAV and multivariate statistical analysis identified seven potential differential OACs. The total fatty acid (FA) contents in different cuts ranged from 262.99 to 3083.98 mg/100 g, and 18 potential differential FAs were identified. Correlation analysis further indicated that FAs were broadly associated with lipid-oxidation-related OACs, whereas the associations between OACs and specific sensory attributes are more concentrated and exhibit attribute-specific differences. The study results reveal flavor differences among different pork cuts from sensory characteristics, OACs, and FAs, providing a theoretical basis for the evaluation of flavor quality and the differentiated processing and utilization of pork cuts.

## 1. Introduction

Pork is an important source of animal-derived food in the diet of Chinese residents. As consumer demand has shifted from quantity to quality, pork quality evaluation has gradually expanded from traditional indicators such as growth performance, carcass traits, and basic nutritional composition to more comprehensive attributes, including flavor, nutritional value, textural properties, and consumer acceptance [1,2]. Aroma is an important sensory dimension in the evaluation of pork flavor characteristics [3]. The chemical basis of pork aroma mainly arises from volatile organic compounds (VOCs) formed during thermal processing or oxidative reactions, including aldehydes, alcohols, ketones, esters, furans, and sulfur- and nitrogen-containing compounds [4]. Because odorants differ greatly in odor thresholds and sensory potency, compounds present at relatively low concentrations may contribute substantially to overall aroma, whereas abundant volatiles may have little perceptual relevance [5]. Therefore, characterization of pork aroma requires particular attention to odor-active compounds (OACs) that are perceived and contribute to sensory expression, rather than relying solely on differences in total volatile profiles [6].

Different cuts are not homogeneous meat matrices. They differ in muscle fiber type, intramuscular lipid composition, connective tissue proportion, energy metabolism pattern, and the extent of adipose tissue deposition. These differences can further influence the extent of lipid oxidation, the availability of lipid-derived aroma precursors, and the generation of odorants [7]. In particular, muscle fiber type is associated with differences in intramuscular lipid composition, especially the phospholipid fraction, which is relatively rich in polyunsaturated fatty acids and susceptible to oxidation. In contrast, total adipose lipid content mainly reflects the extent of fat deposition and the abundance of neutral storage lipids [7,8]. Previous studies have shown that differences among pork cuts are accompanied by variations in fatty acid composition, which provides an important substrate basis for interpreting cut-related aroma variation [8,9]. Wood et al. reported that differences in fatty acid composition among muscles are associated with muscle fiber type. ‘‘Red’’ muscles generally contain a higher proportion of phospholipids and polyunsaturated fatty acids, and thus are more likely to develop differential lipid-oxidation-derived flavor [10]. Thus, pork cuts should not be regarded simply as different sampling locations; rather, differences in both intramuscular lipid composition and adipose lipid abundance contribute to distinct precursor environments for aroma-active compound formation.

Recent studies have increasingly demonstrated this cut-dependent chemical heterogeneity. Gas chromatography–ion mobility spectrometry (GC-IMS) profiling has revealed distinct VOC fingerprints among pork muscles [11], while gas chromatography–olfactometry–mass spectrometry (GC-O-MS) combined with relative odor activity values and phospholipidomics has identified differences in aroma-relevant compounds, fatty acids, and phospholipid precursors among loin, belly, shoulder, and hind-leg samples [9]. Multi-omics investigations have further linked cut-related flavor differences with lipid composition, metabolites, gene expression, and muscle fiber characteristics [7]. More recently, free fatty acids and large-scale VOC profiles have been compared across four pork cuts, with odor activity value (OAV)-based screening and correlation analysis further demonstrating associations between lipid composition and potentially important odorants [12]. Collectively, these studies have established that pork cuts differ substantially in volatile composition and lipid background. However, most have focused primarily on identifying differential VOCs, characteristic compounds, or their potential lipid precursors. A less resolved question is how sensory-relevant odor activity itself is organized across pork cuts. Specifically, it remains unclear whether aroma differentiation among cuts is mainly associated with the occurrence of different high-potency odorants, or whether different cuts largely share the same important odorants but differ in their odor-activity patterns. Moreover, the extent to which such odor-active patterns correspond to directly measured sensory differences among pork cuts remains insufficiently characterized. This distinction is important because chemical differentiation at the VOC level does not necessarily translate directly into perceptual differentiation. Collectively, these studies have established substantial cut-dependent differences in pork volatile and lipid profiles. However, most have focused on differential VOCs or potential precursors, while the sensory relevance of these chemical differences remains less well resolved. Although solvent-assisted flavor evaporation (SAFE) may exhibit compound-dependent recovery because of partitioning between the lipid matrix and extraction solvent, and highly volatile compounds may be partially lost during solvent handling and concentration, it remains well suited for meat products because it enables gentle isolation of a broad range of aroma compounds from complex, lipid-rich matrices [13]. Gas chromatography–olfactometry–quadrupole time-of-flight mass spectrometry (GC-O-QTOF) further combines direct olfactory detection with high-resolution mass-spectrometric identification [14]. Therefore, integrating SAFE-GC-O-QTOF with aroma extract dilution analysis (AEDA) and quantitative analysis provides a sensory-directed approach to determine whether aroma differentiation among pork cuts reflects differences in key odorant identity or in the abundance patterns of shared OACs.

In the present study, five representative pork cuts—pork belly (PB), pork ham (PH), pork shank (PS), pork loin (PL), and pork shoulder butt (PSB)—were systematically investigated using quantitative descriptive sensory analysis, SAFE-GC-O-QTOF, AEDA, quantitative analysis, and fatty acid profiling. The objectives of this study were to: (1) characterize the sensory profiles and OACs of different pork cuts; (2) identify the major OACs contributing to cut-related aroma differences; (3) determine whether aroma differentiation among cuts is associated primarily with differences in odorant identity or with differences in the abundance patterns of shared OACs; and (4) examine the relationships among OAC profiles, fatty acid composition, and sensory characteristics across pork cuts. This study provides a sensory-relevant chemical basis for understanding aroma differentiation among different pork cuts.

## 2. Results and Discussion

### 2.1. Sensory Characteristics of Different Pork Cuts

**Figure 1:**
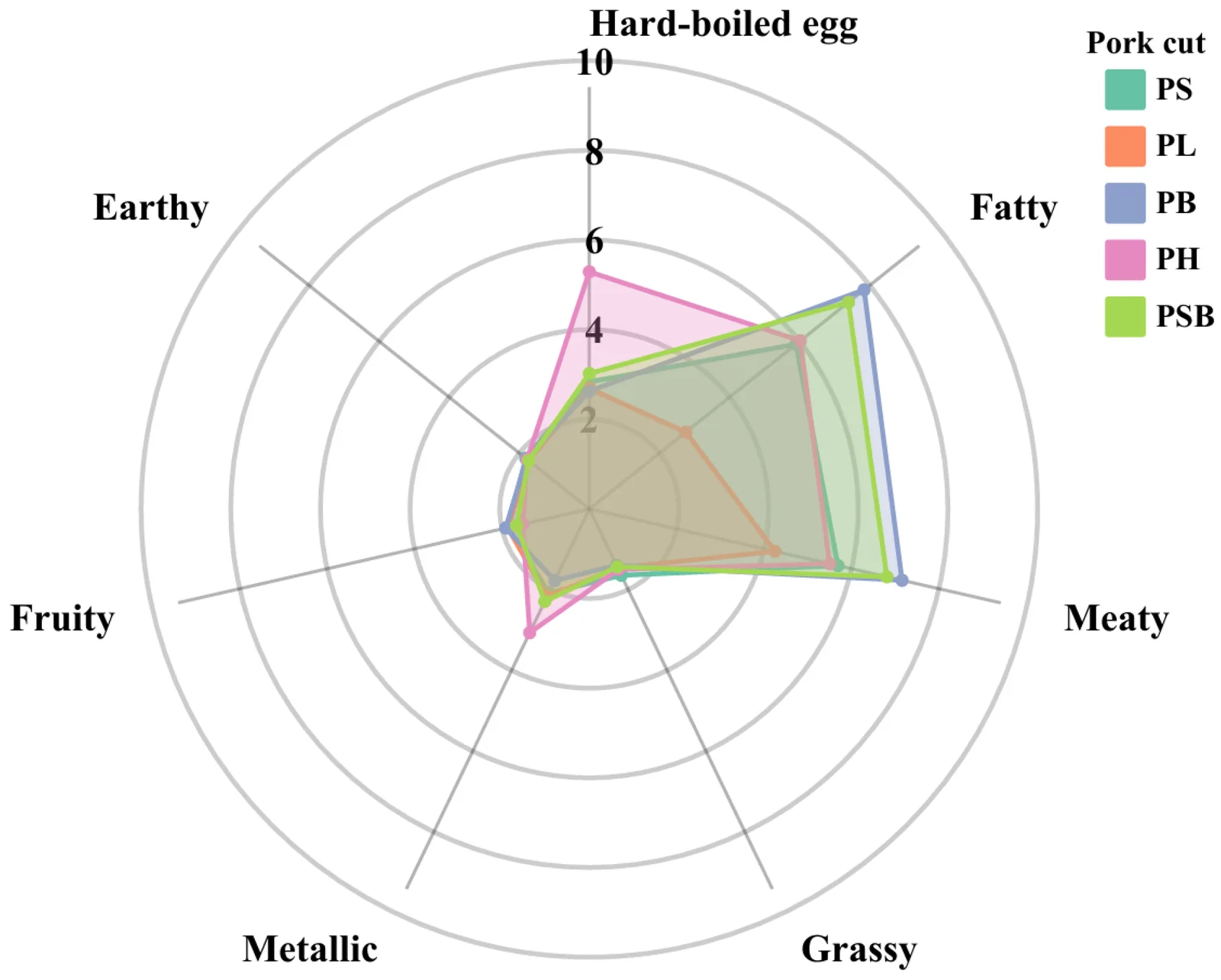
Sensory aroma profiles of five pork cuts based on quantitative descriptive analysis. PB, pork belly; PH, pork ham; PS, pork shank; PL, pork loin; PSB, pork shoulder butt.

As shown in Figure 1, the five pork cuts exhibited different sensory aroma profiles, but the overall profiles were characterized primarily by meaty and fatty aromas. PB, PS, and PSB had relatively high scores for meaty and fatty notes, with PB exhibiting particularly prominent scores for both attributes. However, PL exhibited weaker meaty and fatty notes. PH exhibited aroma characteristics distinct from the other cuts, with relatively strong notes of hard-boiled egg and metallic notes. In contrast, the overall intensity of fruity, grassy, and earthy notes was low across all five pork cuts. These results indicate that the sensory differences among various pork cuts are primarily reflected in relative variations in meaty and fatty notes, as well as certain characteristic odor attributes, rather than a synchronized increase or decrease in overall aroma intensity.

Differences in the sensory aroma profiles of the five pork cuts may be attributed to the inherent tissue and lipid characteristics of each cut. Previous studies have demonstrated significant variations among pork cuts regarding volatile compound composition, lipid profiles, and muscle fiber characteristics; these differences influence the availability of aroma precursors and the subsequent formation of volatile compounds [7,9,11]. Notably, PB typically exhibits higher levels of fat deposition; the resulting abundance of lipid substrates likely facilitates the formation of aroma compounds associated with lipid oxidation, consistent with the strong fatty and meaty sensory notes observed for PB in this study [7,9]. In contrast, PH exhibited pronounced hard-boiled egg and metallic notes, representing a sensory profile distinct from those of the other pork cuts. As sensory evaluation alone cannot pinpoint the specific chemical basis for these differences, specifically, whether they arise from the presence of distinct OACs or from variations in the concentration and odor activity of shared OACs, further research is required to elucidate the molecular basis of the aroma differences among these pork cuts.

### 2.2. Composition and Odor Potency of OACs in Different Pork Cuts

**Figure 2:**
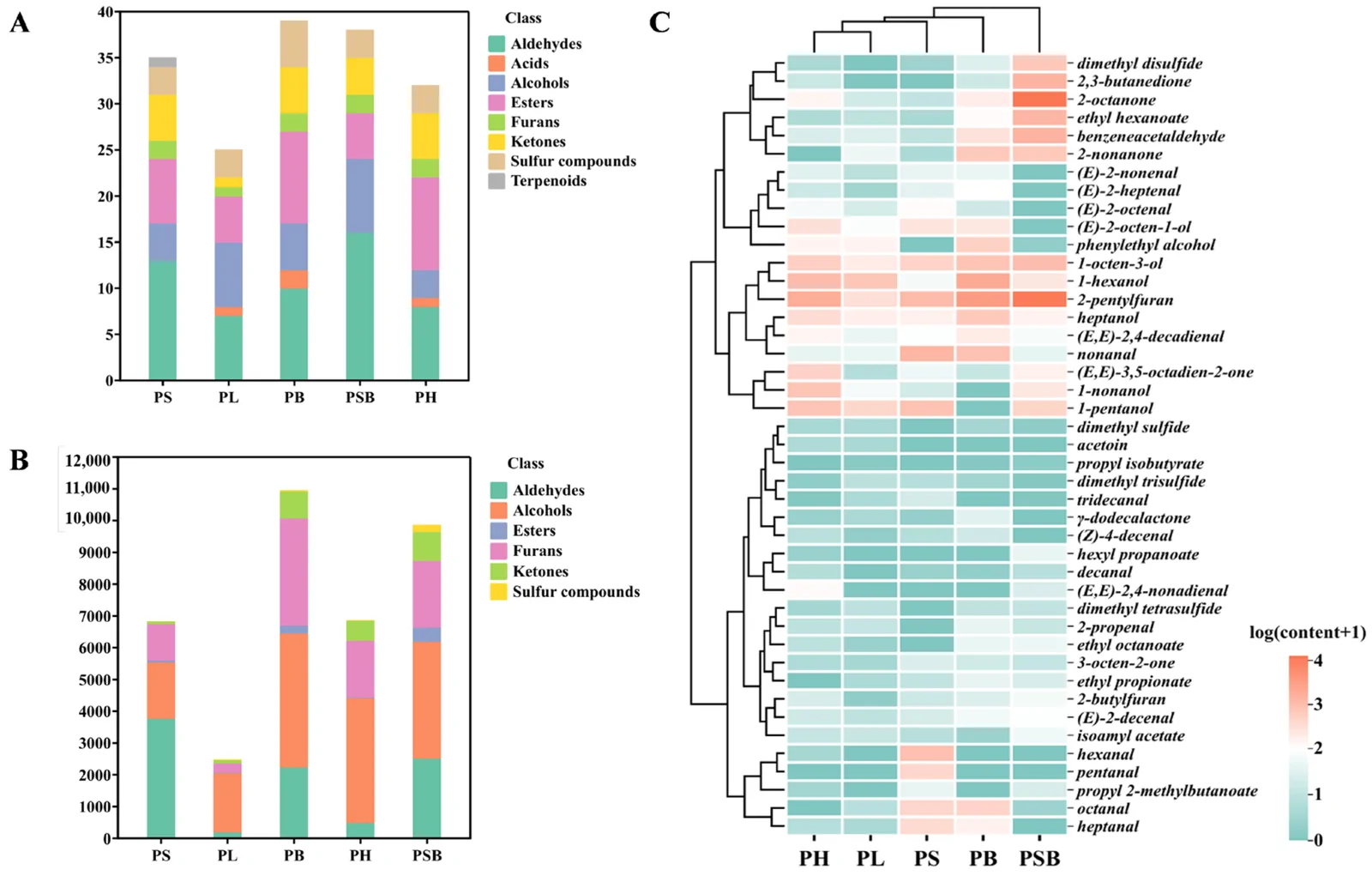
Composition and quantitative abundance patterns of odor-active compounds in five pork cuts. (**A**) Chemical-class composition of OACs identified in the five pork cuts. (**B**) Total concentrations of quantified OACs grouped by chemical class. (**C**) Heatmap showing the abundance patterns of quantified OACs across the five pork cuts. PB, pork belly; PH, pork ham; PS, pork shank; PL, pork loin; PSB, pork shoulder butt.

A total of 62 OACs were identified using SAFE-GC-O-QTOF combined with AEDA, comprising 20 aldehydes, 15 esters, 9 ketones, 8 alcohols, 5 sulfur-containing compounds, 2 furans, 2 acids, and 1 terpene (Table 1). The number of OACs detected varied across the five pork cuts: 39 were identified in PB, while 38, 35, 32, and 25 were detected in PSB, PS, PH, and PL, respectively (Figure 2A). Regarding chemical classes, aldehydes constituted a significant proportion in all cuts, with 16 and 13 types detected in PSB and PS, and 10, 8, and 7 in PB, PH, and PL, respectively. Ten esters were detected in both PB and PH, whereas 7, 5, and 5 were found in PS, PSB, and PL, respectively; alcohols were relatively abundant in PSB and PL, with 8 and 7 types identified, respectively. Additionally, 5 sulfur-containing compounds were detected in PB, compared to 3 in each of the other four cuts. Overall, aldehydes, alcohols, and esters formed the primary chemical composition of the OACs across the five pork cuts. Aldehydes and certain alcohols are primarily associated with lipid oxidation; notably, some unsaturated aldehydes possess low odor thresholds and represent a crucial class of volatile odorants in the formation of cooked meaty aroma [15].

The AEDA results showed that the flavor dilution (FD) values of the identified OACs ranged from 1 to 4096. Eight OACs were detected across the five pork cuts, including benzeneacetaldehyde, nonanal, (*E,E*)-2,4-decadienal, 1-hexanol, 1-octen-3-ol, isoamyl acetate, 2-pentylfuran, and dimethyl trisulfide. Notably, (*E,E*)-2,4-decadienal exhibited FD values of 128–1024 across the five cuts, followed by 1-octen-3-ol (128–512), 2-pentylfuran (64–512), and nonanal (16–512), indicating that these compounds possess high dilution-based olfactory activity within the aroma extracts. These compounds have frequently been reported as key OACs in previous studies on pork aroma. For instance, Chang et al. analyzed pork broth using SAFE-AEDA and confirmed that 1-octen-3-ol, (*E*)-2-octenal, (*E*)-2-decenal, and (*E,E*)-2,4-decadienal were important aroma compounds [16]. Similarly, Han et al. identified OACs with high OAVs, such as hexanal, nonanal, 1-octen-3-ol, heptanal, and 2-pentylfuran, in cooked pork from various breeds [17]. These findings align well with the high FD values observed for the various aldehydes, alcohols, and furans in the present study.

The composition and intensity of high-FD compounds varied markedly across the different pork cuts. In PB, the FD value of (Z)-4-decenal reached 4096 (the highest among all samples) while (*E,E*)-2,4-decadienal reached 1024, and nonanal, 1-octen-3-ol, and 2-pentylfuran all reached 512. These high-FD compounds are primarily associated with odor attributes such as fatty, roasted potato, mushroom, and metallic-like notes, indicating that the OACs with high olfactory activity in PB encompass a diverse range of odor types. In PS, the FD values for both (Z)-4-decenal and (*E,E*)-2,4-decadienal were 1024, with 1-octen-3-ol at 512 and propyl 2-methylbutanoate reaching 256. Unlike the aforementioned high-FD compounds primarily derived from lipid oxidation, propyl 2-methylbutanoate possesses a distinct fruity odor, thereby introducing a prominent ester-like odor characteristic to the high-FD OAC profile of PS. PSB was characterized by (Z)-4-decenal, (*E,E*)-2,4-decadienal, 1-octen-3-ol, and 2-pentylfuran as its primary high-FD compounds. In PH, the FD values for 1-octen-3-ol and 2-pentylfuran were 256, while those for benzeneacetaldehyde and (*E,E*)-2,4-decadienal were 128; notably, benzeneacetaldehyde contributed a sweet odor attribute to the high-FD OACs in PH. Although fewer types of OACs were detected in PL, the FD values for (*Z*)-4-decenal and (*E,E*)-2,4-decadienal still reached 256, with 1-octen-3-ol at 128, and nonanal, 2-pentylfuran, and dimethyl trisulfide all at 64; among these, dimethyl trisulfide exhibited a distinct sulfurous/fishy odor. Overall, the high-FD OACs across the various cuts are primarily aldehydes, alcohols, and furans; notably, lipid-oxidation-related compounds such as 1-octen-3-ol, 2-pentylfuran, and (*E,E*)-2,4-decadienal have also demonstrated high odor activity in studies regarding the aroma of pork and pork fat [6,18]. Meanwhile, although esters and sulfur-containing compounds are fewer in number, they exhibit high FD values in certain cuts and contribute distinct aroma characteristics, such as fruity and sulfur-related notes. Sulfur-containing compounds, in particular, typically possess low odor thresholds, meaning they can elicit strong sensory effects even at trace levels [19]. Yao et al. also found that sulfur-containing compounds like dimethyl disulfide and dimethyl trisulfide are closely associated with the meaty aroma attribute in braised pork [6]. Therefore, the differences in high-FD OACs among various pork cuts are reflected not only in variations in odor activity intensity but also in the compositional differences of compounds representing distinct aroma types.

### 2.3. Multivariate Differentiation and Potential Differential OACs Among Pork Cuts

Based on the AEDA results, 43 OACs with FD ≥ 4 in at least one pork cut were selected for accurate quantification, and their OAVs were subsequently calculated (Table 2). The coefficients of determination (R^2^) for the calibration curves of the target compounds ranged from 0.9831 to 0.9983, indicating good linearity in the quantitative analysis. Marked differences were observed in the total quantitative levels of OACs across the five pork cuts: PB and PSB exhibited the highest levels (10,954.53 and 9873.86 μg/kg, respectively); PH and PS showed intermediate levels (6868.65 and 6833.65 μg/kg, respectively); and PL had the lowest level (2471.47 μg/kg) (Figure 2B). However, the high total OAC levels in PB and PSB were not driven by the same compounds. PB was characterized by high contents of 2-pentylfuran, 1-octen-3-ol, and nonanal, whereas PSB (in addition to 2-pentylfuran) showed high levels of 2-octanone and benzeneacetaldehyde. In PS, nonanal was particularly prominent, reaching 1316.95 μg/kg. Thus, the quantitative differences among the cuts were reflected not only in the variation in total OAC levels but also in the composition and relative abundance of the major OACs (Figure 2C).

The OAV results further indicate that there is no simple correspondence between the quantitative levels of OACs and their potential sensory importance. 2-Pentylfuran was present at high concentrations across all cuts, with OAVs of 292, 185, 498, 555, and 49 in PH, PS, PSB, PB, and PL, respectively; in contrast, (*Z*)-4-decenal was present at lower concentrations, but due to its extremely low odor threshold (0.004 μg/kg), it exhibited OAVs of 1812, 1500, 4232, and 245 in PH, PS, PB, and PL, respectively. Similarly, nonanal showed OAVs of 1317 and 877 in PS and PB, while (*E,E*)-2,4-decadienal displayed high OAVs (237–943) across all five cuts, and 1-octen-3-ol showed OAVs ranging from 192 to 759. These results demonstrate that high abundance does not necessarily equate to high odor activity; OACs with low thresholds can exhibit high OAVs even when present at relatively low concentrations. Therefore, the potential sensory impact of OACs cannot be inferred from concentration alone, but also depends on odor threshold and the compound-specific relationship between concentration and perceived intensity, as dilution may produce only limited changes in sensory intensity for some odorants.

**Figure 3:**
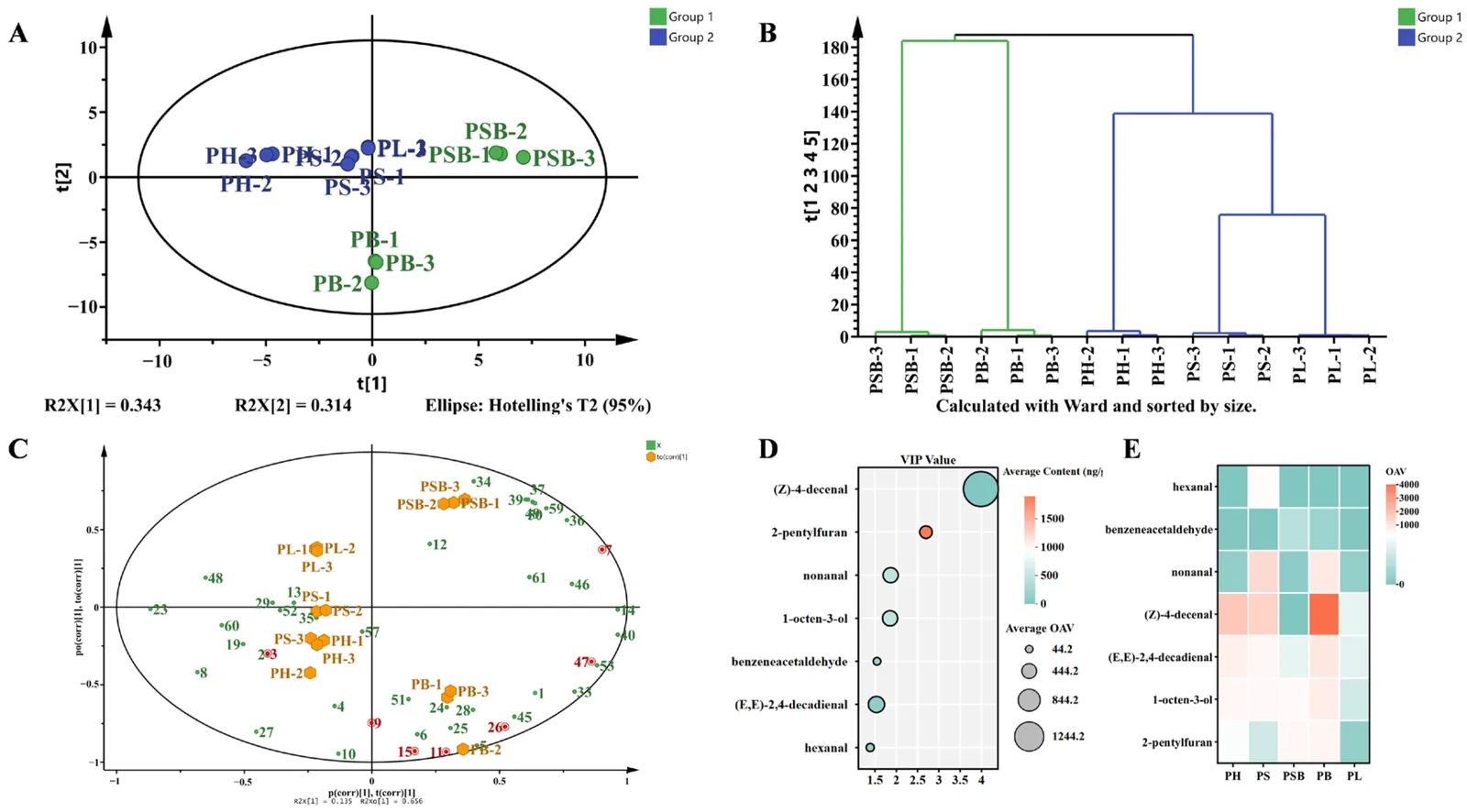
Multivariate analysis of odor-active compound profiles and identification of potential differential OACs among five pork cuts. (**A**) PCA score plot showing the overall distribution of OAC profiles among the five pork cuts. (**B**) Hierarchical clustering analysis of pork cuts based on OAC composition. (**C**) OPLS-DA loading plot showing the contribution of individual OACs to cut differentiation. (**D**) VIP scores of potential differential OACs, with bubble size representing average OAV and color indicating average concentration. (**E**) Heatmap showing the abundance and OAV patterns of the selected potential differential OACs across the five pork cuts. Three independent biological replicates were included for each cut (*n* = 3; 15 observations in total). The OPLS-DA model showed R^2^X = 0.995, R^2^Y = 992, and Q^2^ = 985. PB, pork belly; PH, pork ham; PS, pork shank; PL, pork loin; PSB, pork shoulder butt.

To further compare the overall variations among the 43 quantified OACs, PCA and HCA were performed on the five pork cuts (Figure 3A,B). PC1 and PC2 explained 34.3% and 31.4% of the total variance, respectively, with a cumulative explained variance of 65.7%. In the PCA score plot, PH, PS, and PL were positioned relatively close to one another, whereas PB and PSB were distributed in distinct regions; specifically, PSB was primarily located in the positive direction of PC1, while PB was distributed mainly along the negative direction of PC2. The clustering relationships derived from HCA were generally consistent with the PCA results, further indicating varying degrees of difference in the quantitative OAC profiles among the five pork cuts. Subsequently, OPLS-DA was employed to evaluate the contribution of individual OACs to the differentiation of the cuts (Figure 3C,D). Based on a VIP > 1 criterion, seven potential differential OACs were identified: (*Z*)-4-decenal, 2-pentylfuran, nonanal, 1-octen-3-ol, benzeneacetaldehyde, (*E,E*)-2,4-decadienal, and hexanal. Among these, five were aldehydes, while the remaining two were an alcohol and a furan, respectively. With the exception of benzeneacetaldehyde, most of these compounds are closely associated with lipid oxidation processes, aligning with the important role that lipid oxidation products play in the aroma of cooked pork [15]. Notably, 2-pentylfuran was also identified as a differential volatile organic compound in a study by Duan et al., who compared different pork cuts using GC-IMS and PLS-DA [11]. However, the significantly different volatile flavor compounds identified by Zhang et al. in four pork cuts using untargeted GC-MS and OPLS-DA with rOAV-based screening did not fully align with those found in the present study [12]. Such discrepancies may stem from differences in pig breeds, the selection of cuts, volatile extraction methods, and the scope of screening. As the multivariate analysis in this study utilized OACs confirmed by GC-O as variables, the compounds identified via VIP screening reflect differences in both the quantitative composition and odor-active characteristics of OACs across the various pork cuts.

Further examination of the OAV distribution of these seven potentially differentiating OACs reveals distinct patterns in how they distinguish between different pork cuts (Figure 3E). In PB, compounds such as (*Z*)-4-decenal, nonanal, (*E,E*)-2,4-decadienal, 1-octen-3-ol, and 2-pentylfuran exhibit high OAVs, indicating that the higher total OAC content in PB is accompanied by an enrichment of multiple highly OACs. Notably, several of these aldehydes possess fatty odor characteristics, aligning with the prominent fatty aroma observed in PB. In PS, alongside (*Z*)-4-decenal and nonanal, hexanal, which is characterized by typical green and grassy notes, shows an OAV of 368. This suggests that the differentiation of PS from other cuts also involves variations in the relative odor activity of specific lipid-oxidation-derived aldehydes. In PSB, 2-pentylfuran and benzeneacetaldehyde display relatively high OAVs, indicating a compositional profile of highly odor-active OACs that differs from that of PB. It is worth noting that the seven OACs identified with VIP > 1 do not fully account for all differences observed in sensory evaluation. For instance, while PH exhibits prominent hard-boiled egg and metallic notes, typical sulfur-containing OACs were not among the potentially differentiating OACs with VIP > 1. Sulfur-containing odorants generally have very low odor thresholds and can exert substantial sensory effects even at low concentrations. For example, dimethyl trisulfide remained odor-active in PH with an OAV of 7, despite not being selected as a primary VIP variable. Dimethyl disulfide and dimethyl trisulfide have been associated with characteristic meaty and sulfur-related aromas in cooked pork [6]. More generally, sulfur-containing odorants are characterized by low odor thresholds and high sensory potency [19]. Therefore, the characteristic sensory profile of PH may reflect the contribution of sulfur-containing odorants together with other OACs rather than being explained solely by the seven compounds selected through multivariate discrimination. This demonstrates that OACs contributing significantly to the multivariate differentiation of pork cuts are not necessarily the same compounds that determine specific sensory attributes. While VIP analysis primarily reflects the contribution of OACs to the differentiation of overall quantitative profiles, the specific relationships between sensory attributes and OACs require further evaluation in conjunction with OAV data.

### 2.4. Fatty Acid Profiles and Potential Differential Fatty Acids Among Pork Cuts

**Figure 4:**
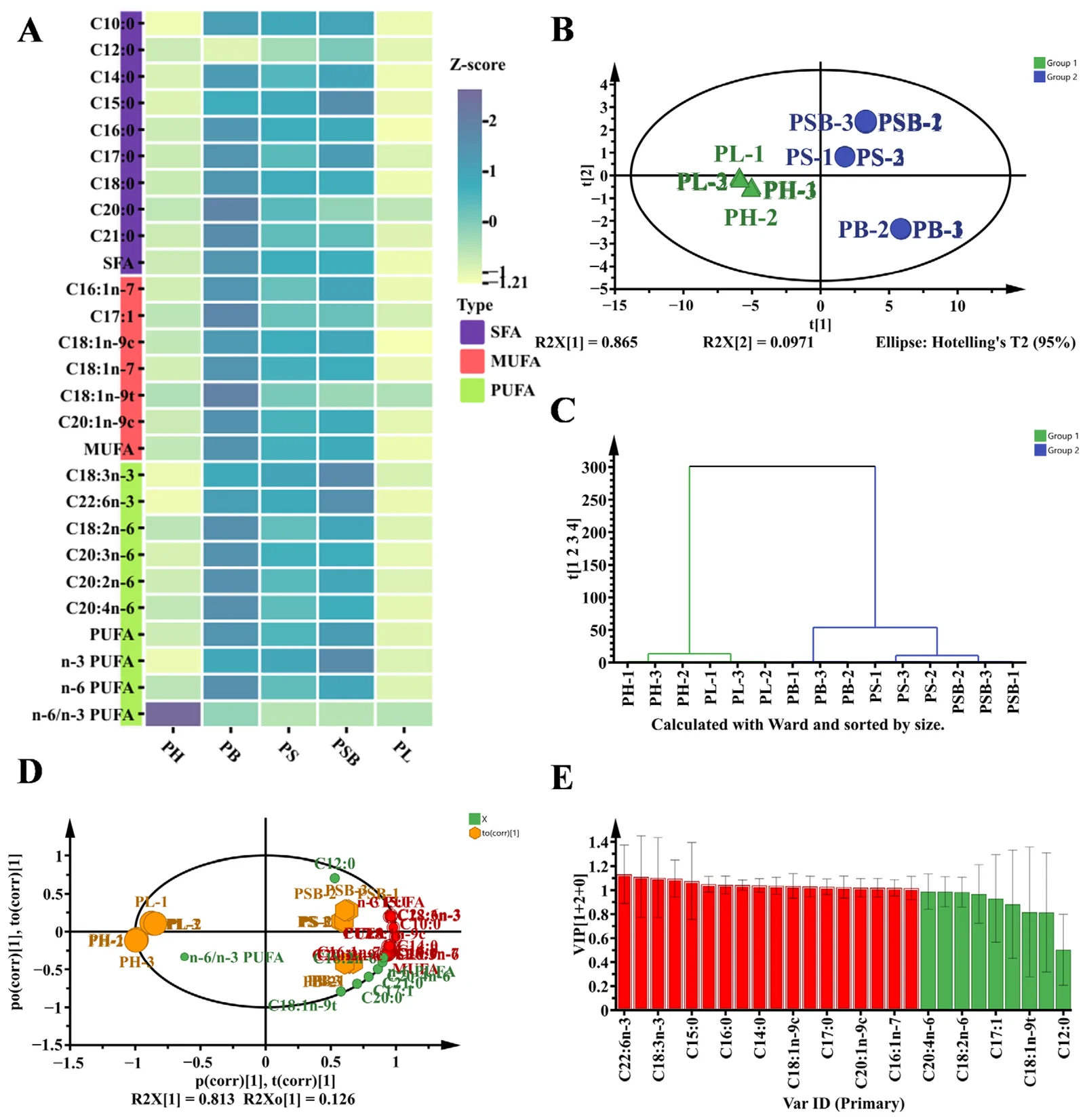
Multivariate analysis of fatty acid profiles and screening of potential differential fatty acids among five pork cuts. (**A**) Heatmap showing the standardized fatty acid profiles of the five pork cuts. (**B**) PCA score plot based on fatty acid profiles. (**C**) Hierarchical clustering analysis of the five pork cuts based on fatty acid composition. (**D**) OPLS-DA loading plot showing the contributions of individual fatty acids to cut differentiation. (**E**) VIP scores of fatty acids for screening potential differential fatty acids among the five pork cuts. Three independent biological replicates were included for each cut (*n* = 3; 15 observations in total). The OPLS-DA model showed R^2^X = 0.957, R^2^Y = 0.975, and Q^2^ = 0.969. PB, pork belly; PH, pork ham; PS, pork shank; PL, pork loin; PSB, pork shoulder butt.

Marked differences in fatty acid content and composition were observed among the five pork cuts (Figure 4A). The complete quantitative fatty acid composition of each cut, including individual fatty acids, SFA, MUFA, PUFA, n-6 PUFA, n-3 PUFA, total fatty acids, and selected fatty acid ratios, is provided in Table S3. While monounsaturated fatty acids (MUFAs) and saturated fatty acids (SFAs) were the predominant fatty acid classes across all cuts, their levels varied considerably. PB exhibited the highest levels of SFA, MUFA, and polyunsaturated fatty acids (PUFA) (1125.96, 1426.77, and 531.25 mg/100 g, respectively), resulting in a total fatty acid content of 3083.98 mg/100 g; PSB followed with a total fatty acid content of 1783.51 mg/100 g. PH and PS showed nearly identical total fatty acid contents (465.53 and 464.37 mg/100 g, respectively), whereas PL had the lowest (262.99 mg/100 g). Among individual fatty acids, C18:1n-9c was abundant in all five cuts, reaching 1199.65 mg/100 g in PB and 739.17 mg/100 g in PSB. C16:0 and C18:0 were the primary SFAs, while C18:2n-6 and C18:3n-3 constituted the major PUFAs, with C18:2n-6 accounting for a large proportion in most cuts. Notably, similar total fatty acid levels do not imply similar specific compositions. For instance, although PH and PS had comparable total fatty acid contents, the C18:3n-3 level in PS was 25.05 mg/100 g compared to only 0.45 mg/100 g in PH; furthermore, their n-6/n-3 PUFA ratios differed markedly, at 1.67 and 13.68, respectively. Thus, variations in fatty acids among different pork cuts involve not only differences in total content but also changes in specific fatty acids and their relative compositions.

Differences in fatty acid composition may further influence the background of lipid-derived aroma precursors across different cuts. Lipids containing unsaturated fatty acids in meat can undergo oxidation during processing, leading to the formation of volatile compounds such as aldehydes, alcohols, ketones, and furans; the fatty acid composition and degree of unsaturation play a crucial role in the formation of these lipid-derived aroma substances [8,20]. In this study, PB exhibited the highest levels of C18:1n-9c (1199.65 mg/100 g) and C18:2n-6 (369.18 mg/100 g), while several OACs commonly associated with lipid oxidation, including nonanal, (*E,E*)-2,4-decadienal, 1-octen-3-ol, and 2-pentylfuran, also exhibited high OAVs. Previous studies have linked nonanal with oleic acid oxidation and hexanal, 2-pentylfuran, and (*E,E*)-2,4-decadienal with linoleic acid oxidation pathways [15]. Thus, the higher levels of unsaturated fatty acids in PB align generally with its richer profile of lipid-oxidation-related OACs. Previous studies on pork have also demonstrated that lipid fractions containing unsaturated fatty acids, such as C18:1, C18:2n-6, and C20:4n-6, are closely linked to the formation of various OACs [9,21]. However, lipid oxidation markers and lipid-class-specific precursors were not directly measured in this study; thus, these results should be interpreted as compositional associations rather than direct evidence of precursor conversion. Moreover, although PH and PS possessed nearly identical total fatty acid levels, they displayed markedly different fatty acid compositions and OAC profiles. This indicates that total fatty acid content alone is insufficient to explain the differences in OACs across cuts; factors such as specific fatty acid composition, the physical state of the lipids, and their oxidative transformation processes must also be considered.

PCA and HCA further revealed overall differences in the fatty acid profiles of the five pork cuts (Figure 4B,C). PC1 and PC2 explained 86.5% and 9.71% of the total variance, respectively, with a cumulative explained variance of 96.21%. PH and PL were primarily distributed along the negative direction of PC1, whereas PB, PS, and PSB were located along the positive direction; PB was further separated from PS and PSB along the PC2 axis. HCA showed similar clustering patterns: PH and PL formed one major branch, while PB, PS, and PSB clustered into another. These results indicate that the differences in fatty acid profiles among the five pork cuts cannot be simply attributed to total fatty acid levels. For instance, although PH and PS had nearly identical total fatty acid contents, they exhibited distinct distributions and clustering patterns in the PCA and HCA analyses; conversely, PS, PB, and PSB, which differed markedly in total fatty acid content, displayed relatively similar overall fatty acid profiles. Therefore, the differences between cuts revealed by multivariate analysis reflect the collective variation in the relative composition of individual fatty acids rather than a simple gradient of fatty acid abundance.

Based on this, OPLS-DA was employed to further evaluate the contribution of different fatty acid variables to sample differentiation (Figure 4D,E). A total of 18 fatty acid variables exhibited VIP values greater than 1, spanning SFA, MUFA, and PUFA categories; notably, C22:6n-3 and C18:3n-3 made substantial contributions to differentiation, while C15:0, C16:0, C14:0, C18:1n-9c, C17:0, C20:1n-9c, and C16:1n-7 also displayed high VIP values. It is worth noting that VIP values do not strictly align with absolute fatty acid content. Although C22:6n-3 is a relatively low-abundance fatty acid, it still showed a high VIP value, indicating that low-abundance fatty acids can also make substantial statistical contributions to sample differentiation. Meanwhile, C18:1n-9c, one of the major fatty acids across the different cuts, also had a VIP > 1, demonstrating that its variation among the cuts contributed strongly to the model’s differentiation capability. Therefore, these 18 fatty acids with VIP > 1 serve as potential differential markers across pork cuts; however, since VIP primarily reflects a variable’s contribution to multivariate differentiation, it does not directly indicate the fatty acid’s importance in aroma formation. In particular, the relationships between specific unsaturated fatty acids, such as C18:1n-9c, C18:2n-6, and C18:3n-3, and specific odor-active compounds require further evaluation through correlation analysis.

### 2.5. Associations Among Sensory Attributes, OACs, and Fatty Acids

**Figure 5:**
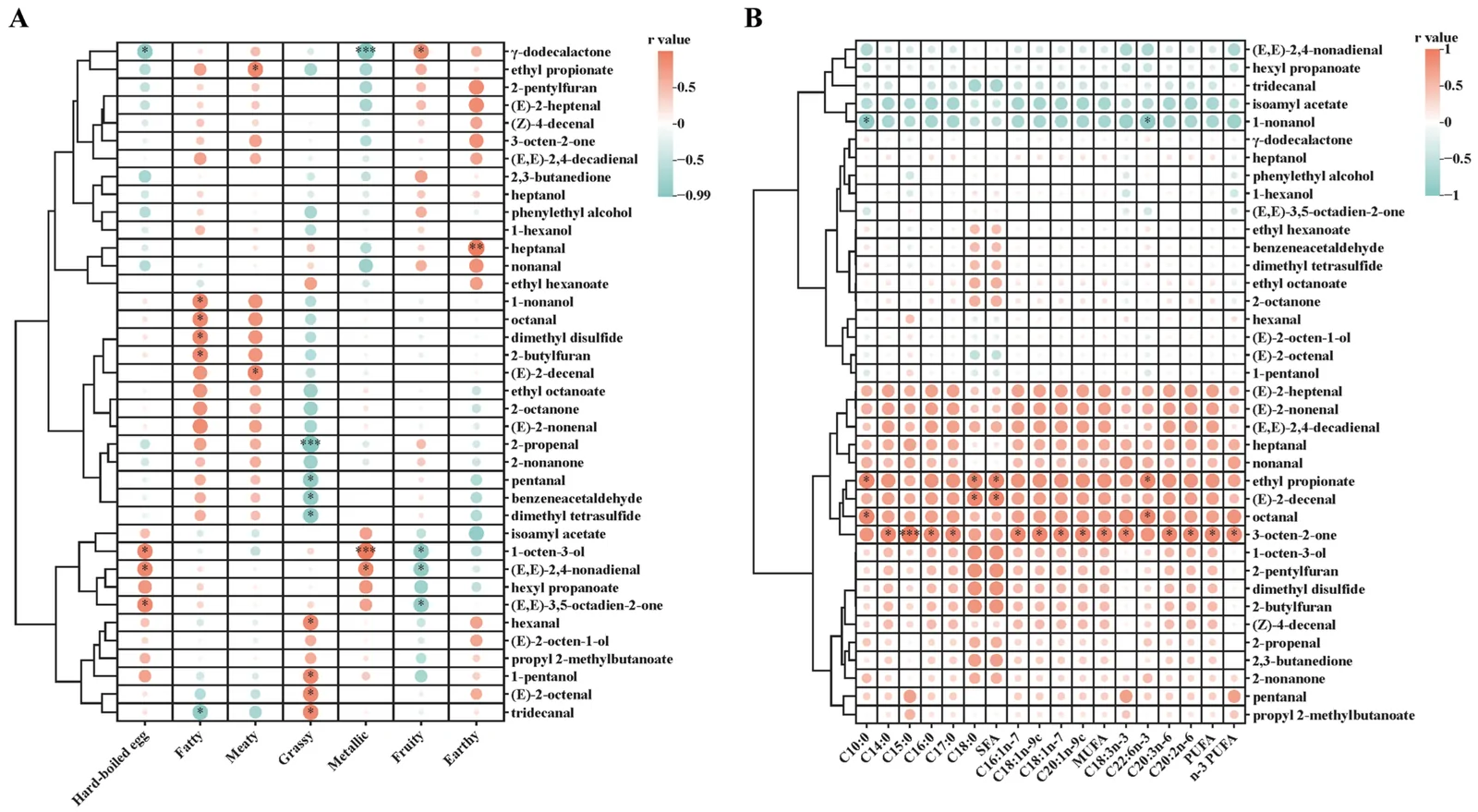
Correlation analysis among sensory attributes, odor-active compounds, and fatty acids in five pork cuts. (**A**) Spearman correlation heatmap between odor-active compounds and sensory aroma attributes. (**B**) Spearman correlation heatmap between fatty acid profiles and odor-active compound concentrations. Three independent biological replicates were included for each cut (*n* = 3; 15 observations in total). Bubble size represents the absolute value of the correlation coefficient, and color indicates the direction and magnitude of the correlation. Asterisks indicate significant correlations (*: *p* < 0.05; **: *p* < 0.01; ***: *p* < 0.001).

Spearman correlation analysis revealed distinct patterns of association between various sensory attributes and OACs (Figure 5A). The fatty attribute showed significant positive correlations with 1-nonanol, octanal, dimethyl disulfide, and 2-butylfuran, whereas the meaty attribute showed significant positive correlations primarily with ethyl propionate and (*E*)-2-decenal. The grassy attribute exhibited mixed associations with various OACs: it was positively correlated with hexanal, 1-pentanol, (*E*)-2-octenal, and tridecanal, but negatively correlated with 2-propenal, pentanal, benzeneacetaldehyde, and dimethyl tetrasulfide. 1-Octen-3-ol showed significant positive correlations with the hard-boiled egg and metallic attributes and a negative correlation with the fruity attribute; (*E,E*)-2,4-nonadienal displayed a similar correlation pattern. These results indicate that a single sensory attribute often co-varies with multiple OACs, while a single OAC may simultaneously correlate with multiple sensory dimensions. Previous studies on pork aroma recombination and omission have similarly demonstrated that overall aroma is typically constituted by a complex array of OACs, and that variations in their relative levels and combinations influence the final sensory profile [3,6,16].

By integrating correlation analysis with the previously mentioned OAV and VIP results, it is possible to further differentiate the odor activity of OACs, their contribution to statistical discrimination, and their relationships with sensory attributes. For instance, while (*Z*)-4-decenal, nonanal, and (*E,E*)-2,4-decadienal exhibited high OAVs in certain cuts, with some also showing VIP values greater than 1, not all of these high-OAV or high-VIP OACs correlated significantly with specific sensory attributes. Conversely, although hexanal did not possess the highest OAV across the cuts, it showed a clear positive correlation with the grassy attribute, consistent with its characteristic green/grassy odor profile. PH exhibited prominent hard-boiled egg and metallic sensory characteristics. Figure 5A reveals that 1-octen-3-ol and certain unsaturated aldehydes were significantly associated with these attributes, demonstrating that the relationship between actual sensory attributes and OACs does not strictly correspond to the odor descriptors of individual compounds. OAV reflects the concentration of a compound relative to its odor threshold, and VIP indicates its contribution to the multivariate differentiation of cuts, whereas correlation analysis reveals the synergistic relationships between OAC variations and specific sensory attributes. These analyses provide complementary descriptive information on the relationships among OAC profiles, fatty acid composition, and sensory attributes.

A distinct correlation pattern was observed between fatty acids and OACs (Figure 5B). Compounds such as (*E*)-2-heptenal, (*E*)-2-nonenal, (*E,E*)-2,4-decadienal, heptanal, nonanal, octanal, 3-octen-2-one, 1-octen-3-ol, and 2-pentylfuran showed overall positive correlations with various fatty acid variables; notably, 3-octen-2-one exhibited strong positive correlations with SFAs, MUFAs, PUFAs, and several individual fatty acids. In contrast, compounds such as 1-nonanol and isoamyl acetate were primarily negatively correlated with most fatty acid variables. Several of the positively associated OACs have previously been linked to lipid oxidation pathways. For instance, the oxidation of C18:1n-9c is closely associated with the formation of nonanal, whereas the oxidation of C18:2n-6 and other PUFAs can yield hexanal, 2-pentylfuran, 1-octen-3-ol, and various unsaturated aldehydes [15,20,21]. Additionally, the broad positive correlations observed in Figure 5B between certain OACs and multiple fatty acid variables may also reflect the influence of concurrent changes in total lipid abundance across the different locations. Consequently, these correlations elucidate the systematic link between variations in fatty acid composition and changes in lipid-oxidation-related OACs, providing evidence to help understand the lipid-chemical basis of aroma formation in different pork cuts.

A comparison of Figure 5A,B reveals that while the correlations between fatty acids and OACs are extensive, the significant correlations between OACs and specific sensory attributes are relatively concentrated and exhibit distinct characteristics. This suggests that variations in fatty acid composition across different pork cuts may influence the formation of various lipid-oxidation-related OACs. However, changes in these OACs are not synchronously reflected across all sensory attributes. Only specific OACs show strong associations with particular sensory attributes, and their sensory impact is jointly determined by factors such as odor thresholds, relative concentrations, and combinations of different OACs. Consequently, flavor differences among pork cuts likely stem from the influence of fatty acid composition on the formation of specific OACs and the varying contributions of these OACs to sensory attributes. These findings also help explain why pork cuts with similar total fatty acid content can still exhibit distinct OAC profiles and sensory characteristics. It is important to note that, because lipid oxidation indices, lipid hydroperoxides, free fatty acids, and phospholipid classes were not directly measured, the involvement of specific fatty acids in the formation of individual OACs remains a mechanistic interpretation based on established pathways rather than direct evidence from the present study. Future studies integrating direct lipid oxidation measurements and lipid-class-specific profiling will be required to verify these precursor–odorant relationships.

## 3. Materials and Methods

### 3.1. Animals and Diets

Healthy Duroc × Landrace × Large White three-way crossbred finishing boars of a new American genetic line were used in this study and were provided by the Tianjin Institute of Industrial Biotechnology, Chinese Academy of Sciences. A total of 30 pigs were included in the present study. The animal protocol was approved by the Animal Ethical and Welfare Committee (AEWC) of Tianjin Xinrui Biotechnology Co., Ltd. (Approval No. XinRui-DWLL-2024011; approved on 16 December 2024). During the feeding period, all pigs were reared under the same husbandry conditions with free access to feed and water. The pigs received a basal diet supplemented with 2% bio-fermented feed additive. The additive was prepared using soybean meal, corn flour, spray-dried corn bran, rice bran, and corn straw as fermentation substrates, accounting for 30%, 20%, 10%, 10%, and 30% of the formulation, respectively. The moisture content of the fermented product was 45%. The composition and nutritional levels of the basal diet are shown in Table S1.

### 3.2. Slaughter and Sample Collection

Slaughter procedures were carried out at a commercial abattoir. The pigs were slaughtered at 158 days of age, with an average slaughter weight of 148.5 kg. Samples were collected within 2 h after slaughter. Five pork cuts, including pork belly (PB), pork ham (PH), pork shank (PS), pork loin (PL), and pork shoulder butt (PSB), were collected from consistent anatomical locations across animals for subsequent analysis. During sample preparation, visible connective tissue and extraneous surface adipose tissue that was not part of the designated cut were removed. Lipid naturally associated with each cut, including intramuscular and cut-associated adipose tissue, was retained to preserve the characteristic tissue composition of each anatomical cut. The prepared samples were then divided according to subsequent analytical use. Samples for sensory evaluation were stored under refrigerated conditions for a short period and analyzed promptly, whereas samples for OAC and fatty acid analyses were rapidly frozen after subdivision and stored at −80 °C until analysis. Repeated freeze–thaw cycles were avoided throughout sample storage and analysis.

### 3.3. Chemicals

All reagents used in this study were of analytical or chromatographic grade. Anhydrous sodium sulfate (≥99.0%), dichloromethane (≥99.8%), and n-alkanes C_7_–C_25_ for linear retention index (RI) calculation were purchased from MREDA Technology Co., Ltd. (Beijing, China). The internal standard 2-methyl-3-heptanone (95.0%GC) was purchased from TCI (Shanghai, China). High-purity nitrogen, helium (99.999%), and liquid nitrogen were supplied by Beijing Tianli Renhe Materials Trading Co., Ltd (Beijing, China). The purchase information of 42 reference standards for qualitative and quantitative analysis of OACs (with serial numbers corresponding to the relevant numbers listed in Table 1, and all had GC purities above 95%.) is as follows: Standards corresponding to numbers 1–9, 14–15, 19, 23–26, 33–37, 51–52, 57, 59, and 61 were purchased from Sigma-Aldrich (St. Louis, MO, USA). Standards corresponding to numbers 10–13, 40, 53, and 60 were purchased from TCI (Shanghai, China). Standards corresponding to numbers 9–10, 42, and 45 were purchased from J&K Scientific (Shanghai, China). Standards corresponding to numbers 28–29, 39, and 45–50 were purchased from Macklin Biochemical Co., Ltd. (Shanghai, China).

### 3.4. Sensory Evaluation

This study was approved by the Ethics Committee of Beijing Technology and Business University under approval number 2025-61. All panelists participated voluntarily after providing informed consent. Quantitative descriptive analysis was used to evaluate the aroma characteristics of pork samples from five anatomical cuts. The sensory panel consisted of 12 trained assessors, including 6 males and 6 females aged 22 to 28 years, recruited from the School of Food and Health, Beijing Technology and Business University. The sensory profiling procedure was developed with reference to the general principles of ISO 13299:2016 for establishing a sensory profile. All panelists had prior experience in food aroma evaluation and received training in aroma recognition, attribute definition, and intensity calibration before the formal experiment. During descriptor generation, panelists first independently evaluated representative pork samples and recorded the odor characteristics they perceived. The generated terms were subsequently discussed by the panel, and synonymous, overlapping, or unclear terms were merged or excluded. Descriptors that were repeatedly perceived across the samples and considered relevant for distinguishing the pork cuts were retained and further compared with aroma attributes previously reported for pork [3,22]. Through this procedure, seven aroma attributes were established: meaty, fatty, fruity, grassy, hard-boiled egg, metallic, and earthy. Before formal evaluation, unified definitions and reference standards were established for each aroma attribute to ensure a consistent understanding of attribute meaning and intensity range among panelists. The reference standards for the seven aroma attributes were as follows: Meaty was defined as cooked meat-like or broth-like aroma and was represented using cooked pork or pork broth; Fatty was defined as a cooked animal fat-like or heated lipid-like aroma and was referenced using heated lipid extracts from pork belly; Fruity was defined as a fruit-like or sweet aroma and was referenced using ethyl hexanoate; Grassy was defined as grass-like or freshly cut grass-like aroma and was referenced using hexanal; Hard-boiled egg was defined as boiled egg-like or heated sulfurous aroma and was referenced using crushed hard-boiled egg; Metallic was defined as a metallic or rust-like odor and was referenced by rust; Earthy was defined as a soil-like or damp soil-like aroma and was referenced using geosmin [23,24,25]. During training, panelists repeatedly evaluated the reference materials and pork samples to become familiar with the attribute definitions and to calibrate their use of the intensity scale. Differences in attribute recognition and intensity scoring were discussed until a consistent interpretation of the descriptors and their intensity ranges was established.

Prior to sensory evaluation, pork samples from each cut were prepared under standardized conditions. Samples from each cut were individually placed in heat-resistant sealed bags and cooked in a water bath at 80 °C until the geometric center temperature reached 72 °C, as monitored using a food thermometer. After cooking, the samples were cut into approximately 2 × 2 × 2 cm cubes. Equal portions were transferred to lidded glass cups and maintained at approximately 55 °C until presentation. All samples were presented within 20 min after cooking to minimize aroma loss, and the lids remained closed until the start of evaluation. Sensory evaluation was conducted in individual booths free of odors and with good ventilation at 25 °C. Samples were labeled with random three-digit codes and presented to panelists in a blind manner. Each aroma attribute was scored on a 0 to 10 intensity scale, where 0 indicated not perceived, and 10 indicated extremely strong intensity. The minimum scoring interval was 0.5. A one min break was required between samples, and coffee beans were used for olfactory recovery. Each sample was evaluated three times, and the final score was calculated as the average of the three evaluations.

### 3.5. Solvent-Assisted Flavor Evaporation (SAFE)

SAFE was used to extract OACs from the pork sample. This method separates volatile components under high vacuum and relatively low temperature, which helps reduce the loss of thermolabile compounds and the formation of artifacts [26]. For each sample, 30.0 g of pork, 60 mL of redistilled dichloromethane, and 60 μL of 2-methyl-3-heptanone (0.816 μg/μL) were added to a 250 mL sealed glass bottle. The mixture was then extracted in a thermostatic shaker at 4 °C and 180 r/min for 8 h. After extraction, the mixture was filtered, and the organic phase was collected. The residue was re-extracted three times with dichloromethane, and all organic phases were combined. The combined organic extract was subjected to distillation in a SAFE apparatus under high vacuum at 1 × 10^−6^ kPa. The temperature of the constant temperature water bath is 40 °C, and the cold trap receiver uses liquid nitrogen to maintain a low-temperature environment for the collect of organic distillate. After the distillate returned to room temperature, anhydrous sodium sulfate was added to remove residual water. The extract was then concentrated to 3 mL using a Vigreux column (50 cm × 1 cm) and further reduced to less than 500 μL under a gentle nitrogen stream. Finally, the extract was adjusted to 1 mL with dichloromethane. Each sample was extracted in triplicate, and the extraction conditions were optimized before the formal experiment.

### 3.6. GC-O-QTOF Analysis

OACs in the SAFE extracts were separated, sniffed, and identified using a GC-O-QTOF system. The system consisted of an Agilent 8890-7250 GC-QTOF (Agilent Technologies, Santa Clara, CA, USA) and an ODP4 olfactory detector (Gerstel GmbH & Co. KG, Mülheim an der Ruhr, Germany). The concentrated SAFE extracts were analyzed separately on a DB-Wax column and a DB-5MS column, both with dimensions of 60 m × 0.25 mm × 0.25 μm (J&W Scientific, Folsom, CA, USA). Samples were injected in splitless mode with an injection volume of 1 μL, and the injector temperature was set at 250 °C. High-purity helium was used as the carrier gas at a flow rate of 1.5 mL/min. The oven temperature program was as follows: the initial temperature was 40 °C and held for 3 min, then increased to 250 °C at 4 °C/min and held for 5 min. Mass spectrometry was performed using an EI ion source at 70 eV with a scan range of m/z 45–500. The transfer line temperature, ion source temperature, and quadrupole temperature were set at 280 °C, 230 °C, and 150 °C, respectively. Each sample was analyzed in triplicate.

GC-O sniffing analysis was carried out by three trained panelists, including two females and one male. Before the formal analysis, all panelists received training in odor recognition and GC-O sniffing procedures. Sniffing analysis was conducted at 25 °C in a well-ventilated environment without obvious airflow disturbance. During the analysis, panelists recorded the retention time, odor description, and odor intensity of each OACs. Odor intensity was rated on a scale from 0 to 3, where 0 indicated not perceived and 3 indicated strong odor. In this study, a volatile compound was classified as odor-active only when it produced a reproducible olfactory response at the GC-O sniffing port, defined as similar odor characteristics perceived by at least two panelists at comparable retention times.

### 3.7. Identification of the OACs

Mass spectral data were processed using Agilent Unknowns Analysis B.10.0 and Agilent MassHunter software (version B.08.00; Agilent Technologies, Inc., Santa Clara, CA, USA). Peak detection was performed using the SureMass algorithm, followed by peak extraction, integration, and compound matching. Compound identification was based on accurate mass calculation combined with library searching and linear retention index information. The matching parameters were set as follows: the maximum number of ions was 20, the minimum relative abundance was 10, the *m*/*z* tolerance was 10 ppm, the peak selection weighting was set to EqualWeight. Candidate OACs were further confirmed by combining mass fragments, accurate mass, linear retention indices, and GC-O odor descriptions. Specifically, the mass spectra of the compounds were compared with the NIST 20 library and the mass spectra of authentic standards. Linear retention indices were calculated by analyzing a mixture of n-alkane standards from C_7_ to C_25_ under the same chromatographic conditions. Odor characteristics were obtained by GC-O analysis and compared with the odor descriptions of authentic standards and literature data [26]. When the mass spectra, linear retention index, and odor characteristics of a compound in the sample were all consistent with those of the corresponding authentic standard compounds, the compound was identified as positively identified. Compounds without authentic standard confirmation but showing general agreement with library and literature data in mass spectra, linear retention index, or odor characteristics were regarded as tentatively identified.

### 3.8. Aroma Extraction Dilution Analysis (AEDA)

AEDA was used to evaluate the contribution of OACs to the overall aroma of different pork samples. The concentrated aroma extracts obtained by SAFE were serially diluted with dichloromethane at a 1:1 volume ratio. Each dilution was analyzed under the same chromatographic conditions as those used for the GC-O analysis described above. Sniffing analysis was performed by three trained panelists. The panelists recorded the retention time and odor description of each OAC until the odor could no longer be perceived at the sniffing port. To maintain nasal moisture and olfactory sensitivity during sniffing, a continuous stream of high-purity gas humidified with water was supplied to the sniffing port. Each sample was analyzed in triplicate. The highest dilution at which an OAC was still perceivable at the sniffing port was defined as its flavor dilution factor (FD). A higher FD value indicates a greater contribution of the OAC to the overall aroma of the sample [27].

### 3.9. Quantitative Analysis and Odor Activity Value (OAV)

Based on the AEDA results, 43 OACs with an FD factor ≥ 4 in at least one of the five pork cuts were selected for quantitative analysis (Table 1). Quantitative analysis was performed using an external standard calibration method with internal standard correction, following the procedure described by Yang et al. [28]. According to the response range and concentration level of each target compound, the corresponding standards were dissolved in dichloromethane to prepare mixed stock solutions and then serially diluted to obtain a series of mixed standard solutions. Because 2-methyl-3-heptanone did not co-elute with all target compounds, it was added to each calibration level as the internal standard (0.816 μg/μL) to compensate for matrix effects and method repeatability errors [29].

All target OACs were analyzed in selected ion monitoring mode. Calibration curves were constructed by plotting the peak area ratio of the target compound to the internal standard (*A_x_*/*A_i_*), against the concentration ratio of the target compound to the internal standard (*C_x_*/*C_i_*), using linear least-squares regression. In this equation, *A_x_* and *C_x_* represent the peak area and concentration of the target compound, respectively, whereas *A_i_* and *C_i_* represent the peak area and concentration of the internal standard. The concentrations of target OACs in the samples were then calculated from the peak area ratios and the corresponding calibration curves. The quantification ions, calibration curves, and correlation coefficients of all target OACs are listed in Table S2. All samples were measured in triplicate, and the results are expressed as mean ± standard deviation. To comprehensively evaluate the contribution of compound concentration and odor threshold to aroma, the OAV of each OAC was further calculated. The OAV was calculated as the ratio of the concentration of a target compound in the sample to its odor threshold, according to the equation, *OAV_i_* = *C_i_*/*OT_i_*, where *C_i_* is the measured concentration of compound i and *OT_i_* is its odor threshold. The sources of odor thresholds are listed in Table S2. Compounds with an OAV greater than or equal to 1 were generally considered to contribute importantly to the overall aroma of the sample [29].

### 3.10. Fatty Acid Analysis

The prepared samples described in Section 3.2, with the characteristic lipid fraction of each cut retained, were thoroughly homogenized before fatty acid analysis. Total lipids were extracted according to the method of Tognocchi et al. with slight modifications [30]. Briefly, 2 g of sample was homogenized with 60 mL of chloroform/methanol solution (2:1, *v*/*v*) and then filtered. The filtrate was mixed with 20 mL of 1 mol/L K_2_CO_3_ solution and incubated at 4 °C for 3 h. The nonpolar phase was then separated and used for fatty acid methyl ester preparation.

Fatty acid methyl esters were prepared according to the method of Kramer et al. using a combined base- and acid-catalyzed methylation procedure with slight modifications [31]. Briefly, 20 mg of extracted lipid was mixed with 1 mL of C19:0 internal standard solution (1 mg/mL), followed by the addition of 0.5 mL of 6% sodium methoxide in methanol. After vortex mixing, the reaction was carried out at 50 °C for 10 min. After cooling to room temperature, 3 mL of HCl/methanol solution (10:90, *v*/*v*) was added, and the mixture was further reacted at 80 °C for 10 min. After cooling again, 2 mL of K_2_CO_3_ solution (6:94, *w*/*v*) and 2 mL of n-hexane were added to extract fatty acid methyl esters. After mixing and centrifugation, the upper hexane phase was collected for gas chromatographic analysis.

Fatty acid methyl esters were analyzed using a 7890-7000C gas chromatography system equipped with a CP-Sil 88 fatty acid methyl ester capillary column (100 m × 0.25 mm × 0.20 μm). A 1 μL aliquot of the fatty acid methyl ester sample was injected with a split ratio of 10:1. The carrier gas flow rate was set at 1.2 mL/min. The injector and detector temperatures were set at 280 °C and 300 °C, respectively. The oven temperature program was as follows: the initial temperature was 100 °C and held for 3 min, then increased to 250 °C at 4 °C/min. Individual fatty acids were identified by comparing their retention times with those of FAME standards (Nu-Chek Prep Inc., Elysian, MN, USA). Quantification was performed using C19:0 as the internal standard, and the results were expressed as mg/100 g meat.

### 3.11. Statistical Analysis

Three independent biological replicates were included for each pork cut (*n* = 3 per cut), and the results are expressed as mean ± standard deviation. Raw data processing and the calculation of means and standard deviations were performed using Microsoft Excel 2024. Radar plots, bar charts, VIP bubble plots, heatmaps, and Spearman correlation analyses were generated using the ChiPlot online platform. Principal component analysis (PCA), hierarchical cluster analysis (HCA), and orthogonal partial least squares discriminant analysis (OPLS-DA) were performed using SIMCA 14.1.

## 4. Conclusions

This study systematically compared the sensory characteristics, OACs, and fatty acid compositions of five pork cuts to elucidate the chemical basis for flavor differences among them. Sensory evaluation revealed distinct aroma profiles for each cut; notably, the PB cut exhibited prominent meaty and fatty aromas, whereas the PH cut displayed strong hard-boiled egg and metallic notes. A total of 62 OACs were identified using SAFE-GC-O-QTOF combined with AEDA, 43 of which were precisely quantified, with total OAC concentrations ranging from 2471.47 to 10,954.53 μg/kg. Based on OAVs and multivariate analysis, seven OACs ((*Z*)-4-decenal, 2-pentylfuran, nonanal, 1-octen-3-ol, benzeneacetaldehyde, (*E,E*)-2,4-decadienal, and hexanal) were identified as potential contributors to the flavor differences. Total fatty acid contents across the cuts ranged from 262.99 to 3083.98 mg/100 g, and 18 fatty acids with VIP scores > 1 were identified as potential differential markers. Correlation analysis revealed extensive associations between fatty acids and various lipid-oxidation-related OACs, whereas significant correlations between OACs and specific sensory attributes were more concentrated. These findings suggest that variations in fatty acid composition influence the formation of certain lipid-oxidation-related OACs, while the ultimate sensory differences are closely linked to the concentration, odor activity, and compositional profile of specific OACs. In summary, this study elucidates the flavor differences among pork cuts across sensory, OAC, and fatty acid dimensions, providing a scientific basis for flavor quality assessment, raw material selection, and differentiated processing and utilization of pork cuts.

## Figures and Tables

**Table 1:** Qualitative, odor description and flavor dilution (FD) factor of odor-active compounds (OACs) in different pork cuts.

No.	OACs	RI ^a^		FD Factors ^b,c^					Odor Description ^d^	**Identification Method ^e^**
		DB-5ms	DB-Wax	PSB	PS	PH	PB	PL		
Aldehydes										
1	2-propenal	<700	828		4				almond	MS/RI/O/STD
2	pentanal	<700	935		2				nutty, cocoa	MS/RI/O/STD
3	hexanal	766	1097	1	128				grassy, green	MS/RI/O/STD
4	heptanal	867	1182	2	8		64	1	green, grassy	MS/RI/O/STD
5	(*E*)-2-heptenal	938	1334	1	2		8		fresh, fatty	MS/RI/O/STD
6	octanal	1000	1291				8		fatty	MS/RI/O/STD
7	benzeneacetaldehyde	1045	1646	8	64	128	64	4	sweety	MS/RI/O/STD
8	(*E*)-2-octenal	1056	1437	16			4	4	green, fatty	MS/RI/O/STD
9	nonanal	1101	1394	32	32	16	512	64	fatty	MS/RI/O/STD
10	(*E*)-2-nonenal	1158	1544	128	128		256	32	green, fatty	MS/RI/O/STD
11	(*Z*)-4-decenal	1191	1542	1024	1024		4096	256	fatty	MS/RI/O/STD
12	decanal	1203	1484	2		4			fatty	MS/RI/O/STD
13	(*E,E*)-2,4-nonadienal	1214	1686	16	4	2			fatty, green	MS/RI/O/STD
14	(*E*)-2-decenal	1259	1590	1	4	2	4		fatty, mushroom	MS/RI/O/STD
15	(*E,E*)-2,4-decadienal	1317	1805	512	1024	128	1024	256	roasted potato	MS/RI/O/STD
16	2-undecenal	1362	1755	1		1			soapy	MS/RI/O
17	2-butyl-2-octenal	1368	1656	2		1			cucumber, fatty	MS/RI/O
18	(*E,Z*)-2,6-dodecadienal	1446	-	1					green	MS/RI/O
19	tridecanal	1507	1824		1				soapy, fresh	MS/RI/O/STD
20	hexadecanal	1815	2137	1					cardboard	MS/RI/O
**Acids**										
21	glycolic acid	<700	-				2	2	buttery	MS/RI/O
22	2-methylpropionic acid	763	1581			2	4		rancid, butter	MS/RI/O
**Alcohols**										
23	1-pentanol	741	1252	2					fusel, sweety	MS/RI/O/STD
24	1-hexanol	828	1360	8	16	1	16	8	green, fruity	MS/RI/O/STD
25	heptanol	953	1467	2	4		4	2	musty, woody	MS/RI/O/STD
26	1-octen-3-ol	967	1458	512	512	256	512	128	mushroom	MS/RI/O/STD
27	(*E*)-2-octen-1-ol	1062	1620	2			2	1	fatty	MS/RI/O/STD
28	phenylethyl alcohol	1113	1912	2			4	2	floral	MS/RI/O/STD
29	1-nonanol	1166	1668	8	4	1		1	fatty, oily	MS/RI/O/STD
30	*τ*-cadinol	1652	2169	2				2	earthy	MS/RI/O
**Esters**										
31	allyl acetate	<700	-		1		2		fruity	MS/RI/O
32	allyl formate	<700	-		2				fruity	MS/RI/O
33	ethyl propionate	702	947			1	4		fruity	MS/RI/O/STD
34	isoamyl acetate	835	1125	4	8	16	4	8	fruity	MS/RI/O/STD
35	propyl 2-methylbutanoate	919	1125	8	256	64			fruity	MS/RI/O/STD
36	ethyl hexanoate	990	1220			8	4		fruity	MS/RI/O/STD
37	propyl isobutyrate	1031	-			4	2	2	fruity	MS/RI/O/STD
38	ethyl 2-hexenoate	1039	1329	1		1	2	2	fruity	MS/RI/O
39	hexyl propanoate	1098	1332		1	4			green, fruity	MS/RI/O/STD
40	ethyl octanoate	1189	1432			2	4		fruity	MS/RI/O/STD
41	amyl caproate	1281	1501				1		sweety, fruity	MS/RI/O
42	furfuryl thiopropionate	1294	-	1	2	2			garlic-like	MS/RI/O
43	octyl isovalerate	1432	-				2	1	floral, honey	MS/RI/O
44	decenyl acetate	1467	1722			2			floral	MS/RI/O
45	*γ*-dodecalactone	1679	2366	1	8		16	2	fatty, buttery	MS/RI/O/STD
**Furans**										
46	2-butylfuran	855	1123	8	4	8	8		fruity, sweet	MS/RI/O/STD
47	2-pentylfuran	983	1229	256	128	256	512	64	metallic-like	MS/RI/O/STD
**Ketones**										
48	acetoin	<700	1273						buttery, creamy	MS/RI/O/STD
49	2,3-butanedione	<700	998			4	1		creamy, buttery	MS/RI/O/STD
50	2-octanone	981	1297	2	2	32	4		dairy, cheesy	MS/RI/O/STD
51	3-octen-2-one	1034	1414		2	1	2		mushroom	MS/RI/O/STD
52	(*E,E*)-3,5-octadien-2-one	1066	1570	4	2				green, grassy	MS/RI/O/STD
53	2-nonanone	1086	1388		2	4	16	1	cheesy, buttery	MS/RI/O/STD
54	2-hydroxy-5-methylacetophenone	1223	2178	2			2		sweet, floral	MS/RI/O
55	1-(4-ethylphenyl)-ethanone	1264	1867	2	1				floral	MS/RI/O
56	2-pentadecanone	1693	2021			2			fatty, fresh	MS/RI/O
**Sulfur compounds**										
57	dimethyl sulfide	<700	755	2	8		2	4	cabbage, sulfur	MS/RI/O/STD
58	1-(methylthio)propane	705	-				2		creamy, green	MS/RI/O
59	dimethyl disulfide	726	1071		32	32	4		onion, cabbage	MS/RI/O/STD
60	dimethyl trisulfide	964	1378	8	1	1	16	64	sulfur, fish	MS/RI/O/STD
61	dimethyl tetrasulfide	1227	1742	2		4	4	4	cabbage, sulfur	MS/RI/O/STD
**Terpenoids**										
62	calamenene	1530	1927		2				herbal, spicy	MS/RI/O

^a^ The retention index (RI) on capillaries DB-5MS and DB-Wax. ^b^ PB: pork belly; PH: pork ham; PS: pork shank; PL: pork loin; PSB: pork shoulder butt. ^c^ The FD factors of all OACs in different pork cuts were determined by AEDA. ^d^ The odor description of each OAC that was sensed on sniffer port of GC-O in different pork cuts. ^e^ The different identification method including mass spectra (MS), retention indices (RI), odor description (O), and standard compounds (STD), respectively.

**Table 2:** Concentrations and odor activity values (OAV) of important odor-active compounds (OACs) in different pork cuts.

**OACs**	**Concentration (μg/kg) and OAV ^a,b^**				
	**PH**	**PS**	**PSB**	**PB**	**PL**
2-propenal	8.46 ± 0.21 (<1)	0.02 ± 0 (<1)	10.12 ± 0.32 (<1)	44.4 ± 2.7 (<1)	11.38 ± 0.67 (<1)
pentanal		417.06 ± 30.7 (34)			
hexanal	3.03 ± 0.18 (1)	884.9 ± 22.64 (368)			
heptanal	6.18 ± 0.37 (2)	354.74 ± 8.59 (118)		158.47 ± 5.66 (52)	3.79 ± 0.11 (1)
(*E*)-2-heptenal	15.96 ± 0.22 (1)	37.77 ± 0.56 (2)		99.52 ± 2.26 (7)	2.37 ± 0.08 (<1)
octanal		420.25 ± 22.09 (8)	1.96 ± 0.16 (<1)	443.96 ± 15.55 (8)	6.73 ± 0.23 (<1)
benzeneacetaldehyde	24.32 ± 0.89 (6)	9.18 ± 0.13 (2)	559.11 ± 36.04 (139)	268.08 ± 19.55 (67)	30.15 ± 2.27 (7)
(*E*)-2-octenal	72.43 ± 4.51 (24)	110.76 ± 0.96 (36)		16.65 ± 1.25 (5)	21.64 ± 0.68 (7)
nonanal	42.92 ± 2.44 (42)	1316.95 ± 111.02 (1317)	30.33 ± 0.84 (30)	877.39 ± 33.03 (877)	49.18 ± 1.93 (49)
(*E*)-2-nonenal	32.43 ± 1.46 (170)	45.87 ± 0.95 (241)		46.95 ± 1.27 (247)	7.67 ± 0.24 (40)
(*Z*)-4-decenal	7.25 ± 0.45 (1812)	6 ± 0.22 (1500)		16.93 ± 0.59 (4232)	0.98 ± 0.05 (245)
decanal	5.6 ± 0.33 (1)	1.37 ± 0.06 (<1)	6.31 ± 0.19 (2)	0.93 ± 0.03 (<1)	
(*E,E*)-2,4-nonadienal	112.62 ± 4.07 (14)		19.08 ± 0.92 (2)		
(*E*)-2-decenal	17.02 ± 1.29 (1)	21.39 ± 0.66 (1)	59.56 ± 3.43 (3)	63.82 ± 5.14 (3)	8.74 ± 0.54 (<1)
(*E,E*)-2,4-decadienal	138.73 ± 9.59 (693)	97.08 ± 1.07 (485)	48.80 ± 1.84 (244)	188.73 ± 6.39 (943)	47.47 ± 1.21 (237)
tridecanal	0.14 ± 0 (<1)	19.93 ± 0.23 (2)	0.12 ± 0.07 (<1)		3.95 ± 0.22 (<1)
1-pentanol	765.43 ± 58.02 (1)	834.81 ± 23.42 (1)	207.62 ± 2.68 (<1)		410.82 ± 10.3 (<1)
1-hexanol	1091.64 ± 46.74 (15)	70.48 ± 3.48 (1)	126.36 ± 4.46 (1)	2053.28 ± 70.65 (29)	756.31 ± 12.45 (10)
heptanol	326.27 ± 12.15 (3)	156.42 ± 8.92 (1)	87.35 ± 1.84 (<1)	671.35 ± 52.52 (7)	176.61 ± 11.6 (1)
1-octen-3-ol	517.08 ± 17.76 (517)	455.51 ± 11.97 (455)	418.25 ± 10.99 (418)	759.86 ± 25.32 (759)	192.65 ± 4.3 (192)
(*E*)-2-octen-1-ol	297.09 ± 11.91 (2)	253.92 ± 19.92 (2)	0.07 ± 0.00 (<1)	226.02 ± 4.56 (2)	88.45 ± 7.62 (<1)
phenylethyl alcohol	145.49 ± 3.81 (1)		1.01 ± 0.03 (<1)	509.69 ± 37.65 (5)	147.57 ± 4.63 (1)
1-nonanol	776.02 ± 18.29 (8)	19.33 ± 1.12 (<1)	119.69 ± 3.46 (1)		71.78 ± 1.73 (<1)
ethyl propionate		9.62 ± 0.15 (<1)	18.19 ± 1.25 (1)	43.73 ± 3.2 (4)	4.21 ± 0.17 (<1)
isoamyl acetate	11.59 ± 0.26 (5)	6.64 ± 0.18 (3)	39.84 ± 2.88 (19)	1.67 ± 0.05 (<1)	11.86 ± 0.7 (5)
propyl 2-methylbutanoate	2.6 ± 0.12 (13)	45.63 ± 1.55 (228)	18.72 ± 1.51 (93)		
ethyl hexanoate	4.31 ± 0.11 (<1)	3.34 ± 0.08 (<1)	506.29 ± 23.62 (14)	110.31 ± 3.91 (3)	8.92 ± 0.22 (<1)
propyl isobutyrate			0.60 ± 0.03 (7)	0.22 ± 0.04 (2)	0.38 ± 0.1 (4)
hexyl propanoate	1.39 ± 0.02 (<1)		31.90 ± 0.65 (3)		
ethyl octanoate	8.38 ± 0.17 (<1)		34.65 ± 1.38 (2)	48.9 ± 2.09 (3)	1.38 ± 0.06 (<1)
*γ*-dodecalactone	1.52 ± 0.08 (<1)	1.04 ± 0.02 (<1)		33.59 ± 1.97 (16)	3.87 ± 0.07 (1)
2-butylfuran	22.17 ± 0.78 (4)	15.86 ± 0.76 (3)	45.06 ± 1.64 (9)	28.34 ± 0.51 (5)	0.77 ± 0.15 (<1)
2-pentylfuran	1757 ± 70.28 (292)	1113.53 ± 57.14 (185)	2988.98 ± 114.34 (498)	3335.79 ± 183.14 (555)	298.54 ± 17.37 (49)
acetoin	4.54 ± 0.24 (<1)				3.52 ± 0.24 (<1)
2,3-butanedione	13.65 ± 1.2 (<1)		545.70 ± 43.80 (36)	15.97 ± 1.18 (1)	
2-octanone	139.25 ± 7.27 (2)	10.25 ± 0.26 (<1)	3239.32 ± 136.81 (64)	180.02 ± 11.44 (3)	17.87 ± 0.86 (<1)
3-octen-2-one	4.67 ± 0.29 (<1)	25.72 ± 0.76 (3)	9.69 ± 0.22 (1)	17.92 ± 0.43 (2)	3.18 ± 0.21 (<1)
(*E,E*)-3,5-octadien-2-one	480.82 ± 6.2 (4)	56.07 ± 2.47 (<1)	90.03 ± 1.80 (<1)	12.58 ± 0.57 (<1)	5.7 ± 0.18 (<1)
2-nonanone		4.01 ± 0.17 (<1)	295.33 ± 14.30 (7)	635.71 ± 60.15 (16)	52.83 ± 2.45 (1)
dimethyl sulfide	3.25 ± 0.27 (3)		0.79 ± 0.03 (<1)	2.8 ± 0.07 (2)	3.73 ± 0.18 (3)
dimethyl disulfide	3.81 ± 0.07 (<1)	1.9 ± 0.08 (<1)	304.06 ± 22.58 (40)	29.32 ± 1.51 (3)	
dimethyl trisulfide	0.72 ± 0.05 (7)	6.3 ± 0.12 (63)	0.19 ± 0.01 (1)	2.38 ± 0.12 (23)	7.92 ± 0.33 (79)
dimethyl tetrasulfide	2.87 ± 0.05 (1)		8.78 ± 0.78 (4)	9.25 ± 0.52 (5)	8.55 ± 0.79 (4)

^a^ Data are expressed as mean concentration ± standard deviation, values in parentheses indicate mean OAV. ^b^ PB: pork belly; PH: pork ham; PS: pork shank; PL: pork loin; PSB: pork shoulder butt.

## Data Availability

The authors declare that all data supporting the findings of this study are available in the paper.

## Supplementary Materials

The following supporting information can be downloaded at: https://www.mdpi.com/article/10.3390/molecules31203642/s1. Table S1. Formulation and nutrient composition of the conventional diet. Table S2. Calibration curves, R² values, odor thresholds (OT), and selected ions of important odor-active compounds (OACs) in different pork cuts [32,33]. Table S3. Fatty acid composition and selected fatty acid ratios of five pork cuts.

## References

[1] Li H.L., Liu Y.Y., Peng Y.L., Yang S.L., Ren H.B., Hu X.G., Zhu J., Deng Y., Cui Q.M., Zhang S.Y. (2024). Comparative characterization and correlation between lipids and volatile organic compounds in NingXiang and Berkshire-Ningxiang pork. Int. J. Food Prop..

[2] Xu S., Zheng W., Zeng Y., Tian X., Xu X. (2025). Analysis of volatile flavor and lipids in different breeds of pork using electronic noses, GC-MS and UPLC-MS/MS. J. Chromatogr. A.

[3] Chen X.L., Liu H.Y., Li C., Xu Y.J., Xu B.C. (2024). Revealing the characteristic aroma and boundary compositions of five pig breeds based on HS-SPME/GC-O-MS, aroma recombination and omission experiments. Food Res. Int..

[4] Wu W.D., Zhan J.L., Tang X.Y., Li T., Duan S.N. (2022). Characterization and identification of pork flavor compounds and their precursors in Chinese indigenous pig breeds by volatile profiling and multivariate analysis. Food Chem..

[5] Regmi A., Ibeogu I.H., Jiang H., Yar M.S., Zhang J., Hou Y., Shan K., Wu J.Q., Li C.B. (2026). Comparative multimodal profiling of flavor signatures in indigenous Chinese pork breeds and muscle cuts using GC-MS, E-nose, and quality attributes. Food Res. Int..

[6] Yao Y., Huang M., Wang X., Yu J., Cui H., Hayat K., Zhang X.M., Ho C.T. (2024). Characteristic volatile compounds contributed to aroma of braised pork and their precursor sources. Food Chem..

[7] Zhang Y., Diao Y., Raza S.H.A., Huang J., Wang H., Tu W., Zhang J., Zhou J., Tan Y. (2025). Flavor characterization of pork cuts in Chalu black pigs using multi-omics analysis. Meat Sci..

[8] Dinh T.T.N., To K.V., Schilling M.W. (2021). Fatty acid composition of meat animals as flavor precursors. Meat Muscle Biol..

[9] Duan S., Tian Z., Zheng X., Tang X., Li W., Huang X. (2024). Characterization of flavour components and identification of lipid flavour precursors in different cuts of pork by phospholipidomics. Food Chem..

[10] Wood J.D., Richardson R.I., Nute G.R., Fisher A.V., Campo M.M., Kasapidou E., Sheard P.R., Enser M. (2004). Effects of fatty acids on meat quality: A review. Meat Sci..

[11] Duan S., Tang X., Li W., Huang X. (2023). Analysis of the differences in volatile organic compounds in different muscles of pork by GC-IMS. Molecules.

[12] Zhang L., Luo W., Zhang Z., Li L., Huang Y. (2026). Correlations between free fatty acids and volatile compounds in four pork cuts from Shanxia long black pigs: Implications for cut-specific flavor generation. Front. Nutr..

[13] Engel W., Bahr W., Schieberle P. (1999). Solvent assisted flavour evaporation—A new and versatile technique for the careful and direct isolation of aroma compounds from complex food matrices. Eur. Food Res. Technol..

[14] Moniruzzaman M., Rodríguez I., Ramil M., Cela R., Sulaiman S., Gan S. (2014). Assessment of gas chromatography time-of-flight accurate mass spectrometry for identification of volatile and semi-volatile compounds in honey. Talanta.

[15] Wang S., Chen H., Sun J., Zhang N., Wang S., Sun B. (2023). Effects of cooking methods on aroma formation in pork: A comprehensive review. Food Chem. X.

[16] Chang Y., Wang S., Chen H., Zhang N., Sun J. (2021). Characterization of the key aroma compounds in pork broth by sensory-directed flavor analysis. J. Food Sci..

[17] Han D., Zhang C.-H., Fauconnier M.-L., Mi S. (2020). Characterization and differentiation of boiled pork from Tibetan, Sanmenxia and Duroc × (*Landrace × Yorkshire*) pigs by volatiles profiling and chemometrics analysis. Food Res. Int..

[18] Gerlach C., Leppert J., Chamarro Santiuste A., Pfeiffer A., Boeker P., Wüst M. (2018). Comparative aroma extract dilution analysis (cAEDA) of fat from tainted boars, castrated male pigs, and female pigs. J. Agric. Food Chem..

[19] Li T., Lang Y., Huo Y., Ren X., Cao T., Long Y., Wu B., Huang M., Sun B., Wu J. (2026). Relationship between sulfur compounds and Baijiu flavor: Composition, flavor characteristics, and modulation. Food Chem..

[20] Fu Y., Cao S., Yang L., Li Z. (2022). Flavor formation based on lipid in meat and meat products: A review. J. Food Biochem..

[21] Tian Z., Duan S., Huang X., Tang X., Li W., Zheng X., Li J. (2026). Characterization of volatile components and identification of aroma precursors in different types of pork by multi-omics. Food Chem..

[22] Liu J.M., Huang F., Han D., Qi C.C., Yang P., Zhang C.H., Blecker C. (2025). Mechanisms underlying tomato-mediated suppression of warmed-over flavor (WOF) and flavor enrichment in precooked stewed beef. Food Chem..

[23] Wang Z., Nie T., Gao Y., Zhang H., Chen H., Wang S., Sun B. (2025). Decoding the flavor of traditional Chinese stir-fried pork cuisine: Development of a sensory wheel and exploration of the relationship between sensory attributes and aroma compounds. Food Res. Int..

[24] Chong P.H., Choy J.Y.M., Theng H.P.A., Chiang J.H., Teo P.S. (2026). Sensory profiles and consumer acceptance of plant-based ‘meaty’ sauces derived from yeast-flavourzyme hydrolysates via Maillard reaction. Future Foods.

[25] Niu Y.W., Wu M.L., Xiao Z.B., Chen F., Zhu J.C., Zhu G.Y. (2016). Effect of fatty acids profile with thermal oxidation of chicken fat on characteristic aroma of chicken flavors assessed by gas chromatography mass spectrometry and descriptive sensory analysis. Food Sci. Technol. Res..

[26] Yu M., Li T., Wan S., Song H., Zhang Y., Raza A., Wang C.M., Wang H.Q., Wang H.W. (2023). Sensory-directed establishment of sensory wheel and characterization of key aroma-active compounds for spicy tallow hot pot seasoning. Food Chem..

[27] Song H., Liu J. (2018). GC-O-MS technique and its applications in food flavor analysis. Food Res. Int..

[28] Yang P., Yu M.G., Song H.L., Xu Y.Q., Lin Y.P., Granvogl M. (2022). Characterization of key aroma-active compounds in rough and moderate fire Rougui Wuyi rock tea (*Camellia sinensis*) by sensory-directed flavor analysis and elucidation of the influences of roasting on aroma. J. Agric. Food Chem..

[29] Zhao M., Ma H.R., Hou Y.Q., Li J., Zou T.T., Zhang D.J., Wen R., Li H.L., Song H.L. (2022). Characterization of key odor-active off-flavor compounds in aged pasteurized yogurt by sensory-directed flavor analysis. J. Agric. Food Chem..

[30] Tognocchi M., Conte G., Mantino A., Foggi G., Casarosa L., Tinagli S., Turini L., Scicutella F., Mele M., Serra A. (2023). Linseed supplementation in the diet of fattening pigs: Effect on the fatty acid profile of different pork cuts. Meat Sci..

[31] Kramer J.K., Fellner V., Dugan M.E., Sauer F.D., Mossoba M.M., Yurawecz M.P. (1997). Evaluating acid and base catalysts in the methylation of milk and rumen fatty acids with special emphasis on conjugated dienes and total trans fatty acids. Lipids.

[32] Van Gemert L.J. (2018). Compilations of Odour Threshold Values in Air, Water and Other Media.

[33] Pu D.D., Shan Y.M., Zhang L.L., Sun B.G., Zhang Y.Y. (2022). Identification and inhibition of the key off-odorants in duck broth by means of the sensomics approach and binary odor mixture. J. Agric. Food Chem..

