# Supplementary material for: Comparative Characterization of Odor-Active Compounds and Fatty Acids in Five Pork Cuts
Source: Molecules. 2026 Oct 12;31(20):3642. doi: 10.3390/molecules31203642 (PMC13707980; doi:10.3390/molecules31203642)
Supplement: Supplementary file 1 [file molecules-31-03642-s001.zip › molecules-4615019-supplementary.pdf]

Supplementary Materials

Comparative Characterization of Odor-Active Compounds and Fatty Acids in Five Pork Cuts

Mingguang Yu [1], Ting Li [2], Liran Wang [3], Liang Zhuang [4,*] and Yunxia Luan [1,*]

[1] Institute of Quality Standard and Testing Technology, Beijing Academy of Agriculture and Forestry Sciences, Beijing 100097, China; a1402010411@163.com
[2] Key Laboratory of Geriatric Nutrition and Health, Ministry of Education, Beijing Technology and Business University, Beijing 100048, China; liting120902@163.com
[3] Zhongke Yuansheng Biotechnology (Tianjin) Co., Ltd., Tianjin 300450, China; wangliran1990@outlook.com
[4] Institute of Agri-food Processing and Nutrition, Beijing Academy of Agriculture and Forestry Sciences, Beijing 100097, China
* Correspondence: zhuangliang3641@126.com (L.Z.); luanyunxia@163.com (Y.L.)

Table

Table S1. Formulation and nutrient composition of the conventional diet

Category	Item	Content (%)
Ingredients	Expanded soybean meal	55
	Expanded full-fat soybean flour	15.5
	Fish meal	10
	Fermented soybean meal	7.5
	Soy protein concentrate	5.5
	High-quality oil	3
	Dicalcium phosphate ($CaHPO_4$)	1.28
	Calcium carbonate	1.15
	Sodium chloride	0.62
	Choline chloride	0.45
Nutrient composition	Crude protein	42
	Crude fiber	9.5
	Crude ash	18.5
	Crude fat	6.28
	Lysine	3.8
	Methionine	2.3
	Tryptophan	1.1
	Threonine	3.9
	Calcium	2
	Net energy (NE, MJ/kg)	9.37
	Gross energy (MJ/kg)	15.48

Table S2. Calibration curves, R^2 values, odor thresholds (OT), and selected ions of important odor-active compounds (OACs) in different pork cuts

No. [a]	Linear equation	R^2	Selected ions (*m/z*) [b]	OT (μg/kg)
1	y=1.2845x-0.1456	0.9931	55, **56**	110[c]
2	y=0.6843x-0.0078	0.9934	**58**, 86	12[d]
3	y=0.9458x-0.0029	0.9947	**56**, 57, 100	2.4[d]
4	y=0.5846x-0.0061	0.9962	**70**, 81, 114	3[d]
5	y=0.6847x+0.0129	0.9917	55, **83**, 112	13[d]
6	y=0.9347x-0.0068	0.9954	**84**, 110, 128	52[d]
7	y=0.6478x-0.0042	0.9907	**91**, 92, 120	4[c]
8	y=0.8658x-0.0273	0.9919	**70**, 83, 126	3[c]
9	y=0.8235x-0.0154	0.9938	**98**, 114, 142	1[d]
10	y=0.5836x-0.0028	0.9893	**70**, 96, 140	0.19[e]
11	y = 1.2587x-0.0193	0.9975	**84**, 110, 154	0.004[c]
12	y=0.8547x-0.0098	0.9946	**57**, 112, 156	3[d]
13	y=0.6945x-0.0018	0.9954	**81**, 138	8[c]
14	y=0.8543x+0.0048	0.9983	**70**, 83, 154	17[c]

15	y=0.7847x-0.0028	0.9936	**81**, 152	0.2[c]
19	y=1.1854x+0.0172	0.9907	**82**, 96, 198	8[c]
23	y=0.4938x-0.0096	0.9945	**55**, 70, 88	730[d]
24	y=0.6087x-0.1084	0.9831	**56**, 69, 102	70[d]
25	y=0.7834x-0.0052	0.9916	**70**, 116	94[c]
26	y=0.8842x-0.0141	0.9929	**57**, 72, 128	1[c]
27	y=0.9238x-0.0056	0.9941	**57**, 110, 128	100[c]
28	y=0.8235x-0.0112	0.9853	**91**, 92, 122	86[c]
29	y=0.7538x-0.0595	0.9897	**56**, 98, 144	90[c]
33	y=0.9236x-0.0106	0.9849	**57**, 102	10[c]
34	y=1.0894x+0.0092	0.9947	**70**, 130	2[c]
35	y=0.9456x+0.0052	0.9971	85, **103**, 144	0.2[c]
36	y=0.7894x-0.0115	0.9958	**88**, 99, 144	36[c]
37	y=1.0632x+0.0163	0.9831	**71**, 89, 130	0.086[c]
39	y=0.9342x-0.0027	0.9933	**57**, 75, 158	8[c]
40	y=0.8542x-0.0110	0.9942	**88**, 101, 172	15[c]
45	y=1.0735x-0.0143	0.9957	**85**, 198	2[c]
46	y=1.2856x-0.0158	0.9932	**81**, 124	5[c]
47	y=0.8632x-0.0187	0.9914	**81**, 82, 138	6[d]
48	y=0.4153x-0.0096	0.9913	**45,** 88	14[c]
49	y=1.4734x-0.0194	0.9962	**43**, 86	15[c]

50	y=0.9287x-0.0194	0.9891	**58**, 85, 128	50[c]
51	y=1.0946x-0.1087	0.9902	97, **111**, 126	6.7[c]
52	y=0.5268x+0.0024	0.9876	**95**, 109, 124	100[c]
53	y=1.0956x+0.0117	0.9952	58, **71**, 142	38[c]
57	y=1.0894x-0.0114	0.9951	47, 61, **62**	1[d]
59	y=0.9847x-0.0089	0.993	**79**, 94	7.5[d]
60	y=0.9234x-0.0108	0.9922	79, 111, **126**	0.1[c]
61	y=1.0247x-0.0083	0.9967	79, 94, **158**	1.8[c]

[a] The numbering of OACs corresponds to that in the table 1.

[b] Qualitative ions, where the bolded ions are also used for quantification.

[c] Threshold values were obtained from Gemert et al. (2011)[32].

[d] Threshold values were obtained from Chen et al. (2024)[3].

[e] Threshold values were obtained from Pu et al. (2020)[33].

Table S3. Fatty acid composition and selected fatty acid ratios of five pork cuts

Fatty acid	Content (mg/100g)				
	PH	PB	PSB	PS	PL
C10:0	0.37 ± 0.13	4.23 ± 0.27	1.53 ± 0.06	0.54 ± 0.06	0.31 ± 0.11
C12:0	0.49 ± 0.06	2.01 ± 0.17	0.83 ± 0.25	0.75 ± 0.05	0.43 ± 0.03
C14:0	2.37 ± 0.23	41.77 ± 3.47	18.83 ± 0.75	3.49 ± 0.36	1.70 ± 0.28
C14:1	0.24 ± 0.04	1.90 ± 0.20	1.27 ± 0.06	0.26 ± 0.03	0.12 ± 0.03
C15:0	0.47 ± 0.11	7.88 ± 0.58	3.32 ± 0.28	0.68 ± 0.32	0.27 ± 0.15
C16:0	104.05 ± 6.76	614.82 ± 14.41	370.85 ± 21.91	91.01 ± 7.84	61.00 ± 5.31

C16:1n-7	5.37 ± 0.65	88.52 ± 8.77	37.19 ± 1.80	7.37 ± 1.24	4.23 ± 0.45
C17:0	0.62 ± 0.07	11.99 ± 1.74	5.57 ± 0.27	1.14 ± 0.23	0.19 ± 0.08
C17:1	0.46 ± 0.04	13.27 ± 1.41	5.09 ± 0.66	1.25 ± 0.11	0.19 ± 0.03
C18:0	66.07 ± 3.18	357.84 ± 13.51	249.58 ± 10.46	63.05 ± 5.03	35.39 ± 3.16
C18:1n-9c	231.96 ± 11.71	1199.65 ± 71.52	739.17 ± 4.98	210.05 ± 5.84	129.72 ± 11.85
C18:1n-9t	0.32 ± 0.06	80.35 ± 1.69	11.69 ± 1.18	0.41 ± 0.16	0.31 ± 0.10
C18:1n-7	0.87 ± 0.12	10.89 ± 1.03	4.94 ± 0.37	1.27 ± 0.02	0.50 ± 0.13
C18:2n-6	43.35 ± 2.96	369.18 ± 15.25	205.83 ± 12.86	45.03 ± 2.86	8.70 ± 0.22
C18:3n-3	0.44 ± 0.05	107.24 ± 3.41	56.71 ± 5.32	25.05 ± 2.25	15.42 ± 1.57
C20:0	0.33 ± 0.04	74.74 ± 7.14	15.94 ± 1.22	0.52 ± 0.13	0.33 ± 0.07
C20:1n-9c	1.99 ± 0.23	32.20 ± 2.47	19.32 ± 2.06	3.09 ± 0.25	1.05 ± 0.16
C20:2n-6	1.13 ± 0.12	17.93 ± 1.03	10.43 ± 0.84	1.76 ± 0.21	0.56 ± 0.17
C20:3n-6	0.83 ± 0.07	9.52 ± 0.99	5.04 ± 0.43	1.46 ± 0.08	0.51 ± 0.24
C20:4n-6	0.45 ± 0.04	9.19 ± 0.10	3.65 ± 0.15	0.63 ± 0.21	0.29 ± 0.05
C21:0	0.49 ± 0.05	10.70 ± 1.07	4.47 ± 0.53	1.39 ± 0.11	0.40 ± 0.10
C22:6n-3	2.90 ± 0.25	18.20 ± 1.98	12.29 ± 0.52	4.22 ± 0.60	1.40 ± 0.18
SFA	175.25 ± 14.58	1125.96 ± 58.07	670.90 ± 22.07	162.54 ± 2.70	100.00 ± 9.59
MUFA	241.19 ± 16.91	1426.77 ± 34.01	818.67 ± 10.24	223.69 ± 8.28	136.12 ± 13.35
PUFA	49.09 ± 2.64	531.25 ± 29.66	293.95 ± 11.76	78.14 ± 5.90	26.87 ± 2.76
n-6 PUFA	45.75 ± 24.14	405.82 ± 21.37	224.95 ± 10.03	48.88 ± 3.05	10.06 ± 1.01
n-3 PUFA	3.34 ± 0.35	125.44 ± 13.29	69.01 ± 5.80	29.27 ± 2.85	16.82 ± 1.75
Total FA	465.53 ± 29.13	3083.98 ± 121.75	1783.51 ± 0.06	464.37 ± 34.88	262.99 ± 24.70
MUFA/SFA	1.38	1.27	1.22	1.38	1.36
PUFA/SFA	0.28	0.47	0.44	0.48	0.27
UFA/SFA	1.66	1.74	1.66	1.86	1.63
n-6/n-3 PUFA	13.7	3.24	3.26	1.67	0.6

Values are expressed as mean ± standard deviation (mg/100 g meat). SFA, saturated fatty acids; MUFA, monounsaturated fatty acids; PUFA, polyunsaturated fatty acids; UFA, unsaturated fatty acids. MUFA/SFA, PUFA/SFA, UFA/SFA, and n-6/n-3 PUFA represent the corresponding fatty acid ratios.
